# Sphingosine kinase 1 is required for TGF-β mediated fibroblast-to-myofibroblast differentiation in ovarian cancer

**DOI:** 10.18632/oncotarget.6703

**Published:** 2015-12-21

**Authors:** Jessica A. Beach, Paul-Joseph P. Aspuria, Dong-Joo Cheon, Kate Lawrenson, Hasmik Agadjanian, Christine S. Walsh, Beth Y. Karlan, Sandra Orsulic

**Affiliations:** ^1^ Women's Cancer Program at the Samuel Oschin Comprehensive Cancer Institute, Los Angeles, CA, USA; ^2^ Graduate Program in Biomedical Science and Translational Medicine, Cedars-Sinai Medical Center, Los Angeles, CA, USA; ^3^ Department of Obstetrics and Gynecology, David Geffen School of Medicine, University of California, Los Angeles, CA, USA

**Keywords:** sphingosine kinase 1 (SPHK1), cancer-associated fibroblast (CAF), transforming growth factor-beta 1 (TGF-β1), sphingosine-1-phosphate (S1P), microenvironment

## Abstract

Sphingosine kinase 1 (SPHK1), the enzyme that produces sphingosine 1 phosphate (S1P), is known to be highly expressed in many cancers. However, the role of SPHK1 in cells of the tumor stroma remains unclear. Here, we show that SPHK1 is highly expressed in the tumor stroma of high-grade serous ovarian cancer (HGSC), and is required for the differentiation and tumor promoting function of cancer-associated fibroblasts (CAFs). Knockout or pharmacological inhibition of SPHK1 in ovarian fibroblasts attenuated TGF-β-induced expression of CAF markers, and reduced their ability to promote ovarian cancer cell migration and invasion in a coculture system. Mechanistically, we determined that SPHK1 mediates TGF-β signaling via the transactivation of S1P receptors (S1PR2 and S1PR3), leading to p38 MAPK phosphorylation. The importance of stromal SPHK1 in tumorigenesis was confirmed *in vivo*, by demonstrating a significant reduction of tumor growth and metastasis in SPHK1 knockout mice. Collectively, these findings demonstrate the potential of SPHK1 inhibition as a novel stroma-targeted therapy in HGSC.

## INTRODUCTION

High-grade serous ovarian carcinoma (HGSC) is the most common type of ovarian cancer and accounts for the majority of disease-related mortality [1, 2]. One of the main reasons for high mortality is that most ovarian cancer patients (∼75%) present with widespread metastasis at initial diagnosis [3, 4]. The metastatic potential of many cancers, including ovarian, is largely dependent on the bidirectional communication between tumor cells and their surrounding microenvironment [5, 6]. Ovarian cancers have a substantial stromal component that can comprise up to 70% of the tumor [7]. A predominant cell type in the tumor stroma is the cancer-associated fibroblast (CAF). CAFs secrete a milieu of growth factors, cytokines, and extracellular matrix (ECM) components, which generate a microenvironment that enhances tumor cell growth, invasion, and metastatic spread [6, 8, 9]. However, the mechanisms by which CAFs develop remain unclear. Identification of the molecular mediators that facilitate CAF formation is critical to the discovery of more effective therapies in HGSC.

Sphingosine kinases (SPHK1 and SPHK2) are homologous isoenzymes that catalyze the phosphorylation of sphingosine to generate the bioactive metabolite sphingosine-1-phosphate (S1P). Accumulating evidence indicates that sphingolipid metabolism is altered in human cancers and contributes to disease progression, metastasis, and the development of chemoresistance [10–12]. Aberrant SPHK1 and SPHK2 activity has been implicated in diverse malignant processes including neoplastic transformation, proliferation, and migration in numerous cancer types [10, 12–16]. Elevated levels of S1P have also been observed in cancer, where S1P was shown to act both as an intracellular second messenger and as a secreted ligand capable of activating a family of S1P-specific cell surface G-protein coupled receptors (S1PR1-5) [11, 17].

Whereas the impact of altered sphingolipid metabolism has been extensively studied in tumor cells, little is known about the role of the sphingosine kinases and S1P-mediated signaling in the adjacent tumor stroma, and more specifically CAFs. The formation of CAFs shares several similarities with the process of tissue fibrosis, which is characterized by the excessive deposition of ECM by activated myofibroblasts [18, 19]. Studies in models of organ fibrosis have provided insight into the potential roles of SPHK1 in tumor stroma. In a model of pulmonary fibrosis, S1P stimulated the differentiation of normal lung fibroblasts to a pro-fibrotic myofibroblasts state characterized by increased ECM deposition [20]. In a model of cardiac fibrosis, SPHK1 expression in fibroblasts was induced by TGF-β1 and fibroblast-specific inhibition of SPHK1 attenuated TGF-β1-induced myofibroblast activation [21]. TGF-β has been implicated in fibroblast activation in a number of different cancer types, including ovarian [8, 22]. Together, these studies suggest that SPHK1 may be a critical mediator of differentiation and TGF-β-induced activation of CAFs.

In the current study, we investigated whether SPHK1 mediates the differentiation of normal ovarian fibroblasts to CAFs, thereby promoting tumorigenesis in ovarian cancer. We confirmed that HGSCs have elevated levels of SPHK1 mRNA, and that SPHK1 is highly expressed by ovarian cancer-associated stroma. Using an *in vitro* coculture model, we found that ovarian cancer cells stimulated the transition of fibroblasts to activated myofibroblasts, and induced stromal SPHK1 expression. We further showed that knockout or pharmacological inhibition of SPHK1 in ovarian fibroblasts limited their activation by both cancer cells and TGF-β1, attenuating their ability to promote tumor cell migration and invasion. In summary, these data indicate that SPHK1 contributes to ovarian cancer's clinical phenotype as a required mediator of CAF formation, and may serve as a viable therapeutic target.

## RESULTS

### SPHK1 is overexpressed in serous ovarian cancer and associated with poor survival

**Figure 1:**
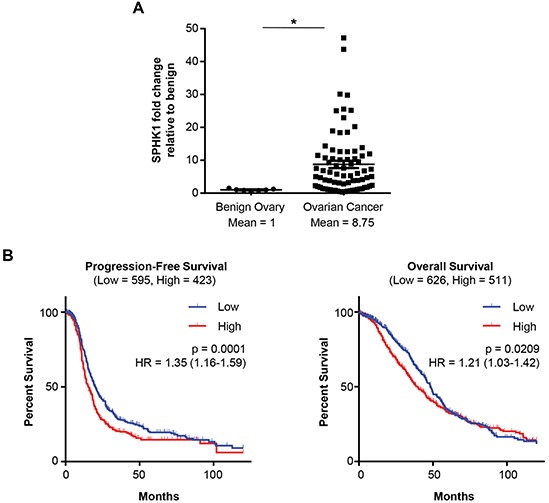
High SPHK1 expression is associated with reduced survival of patients with HGSC **A.** Quantification of SPHK1 mRNA in benign ovaries (*n* = 7) and ovarian cancer (*n* = 77) patient samples by OpenArray Real-Time PCR. Expression levels were normalized to RPLP0. Statistical significance was determined by Mann Whitney *U* test.*p < 0.05. **B.** Kaplan-Meier plot analysis of progression-free and overall survival of patients stratified by SPHK1 transcript levels (Affymetrix ID: 219257_s_at) in a combined cohort of 13 gene expression datasets. Low and high SPHK1 expression were defined by the auto-calculated best cutoff. Significance values were determined by log-rank test. HR indicates the hazard ratio, and “Low” and“High” in parentheses indicate the number of cases per group.

Previous studies have found elevated levels of S1P in the serum and ascites of ovarian cancer patients. Therefore, we hypothesized that expression of SPHK1, the enzyme that produces S1P, would also be altered in ovarian cancer. We observed significantly higher expression of SPHK1 mRNA in the tumor samples compared to the benign ovary controls (p=0.0004) (Figure 1A). In contrast, mRNA levels of SPHK2 were not significantly altered (Supplementary Figure S1A). Publically-available ovarian cancer datasets confirmed elevated SPHK1 mRNA expression in ovarian cancer compared to benign ovary (Bonome dataset) or fallopian tube (the Cancer Genome Atlas [TCGA] dataset) (Supplementary Figure S1B). High SPHK1 expression in tumors was significantly associated with both poor progression-free survival (p = 0.0001) and decreased overall survival (p = 0.0209) (Figure 1B).

### SPHK1 is associated with a reactive stromal signature and is highly expressed by the cancer-associated stroma

**Figure 2:**
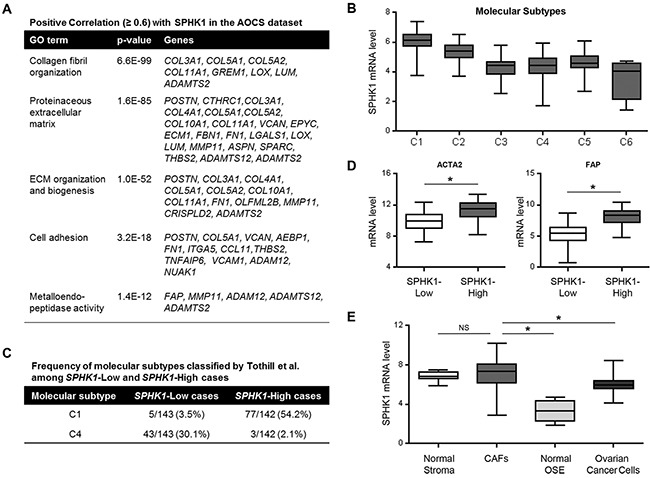
SPHK1 expression is associated with reactive stroma in ovarian cancer **A.** GO enrichment analysis of genes that correlate with SPHK1 expression (Pearson correlation, R ≥ 0.6) in the AOCS dataset (*n* = 285). **B.** SPHK1 transcript levels associated with the classified molecular subtypes of ovarian cancer by Tothill *et al*. in the AOCS dataset. **C.** Frequency of C1 and C4 molecular subtypes among SPHK1-Low and SPHK1-High cases in the AOCS dataset. The C1 molecular subtype is characterized by a reactive stromal signature, while the C4 molecular subtype is described as having a low stromal response signature. Median expression was used to define SPHK1-Low (*n* = 143) and SPHK1-High (*n* = 142) samples. **D.** Box-and-whisker plots of the differences in transcript levels of ACTA2 (encoding αSMA) and FAP, between SPHK1-Low and SPHK1-High tumors in the AOCS dataset. Statistical significance was determined by Mann Whitney *U* test. **E.** Plot showing the expression level of SPHK1 in laser capture-microdissected stromal fibroblastic and epithelial components of both normal and malignant ovarian tissue samples (GSE40595). Statistical significance was determined by Mann Whitney *U* test. In all box-and-whisker plots, horizontal bars indicate the medians, boxes indicate the 25th to 75th percentiles, and whiskers indicate the minimum and maximum values. *p < 0.05. CAFs, cancer-associated fibroblasts; OSE, ovarian surface epithelium.

To identify the biological mechanism that could explain the association of increased expression of SPHK1 and poor survival, we performed gene ontology (GO) enrichment analysis of the genes that positively correlated (R ≥ 0.6) with SPHK1 in the Australian Ovarian Cancer Study (AOCS) and TCGA datasets [23, 24]. Genes involved in collagen fibril organization, ECM production and remodeling, cell adhesion, and metalloendopeptidase (MMP) activity were enriched (Figure 2A and Supplementary Tables S1 and S2).

Tothill et al. classified tumors in the AOCS dataset into six molecular subtypes by their gene expression signatures (C1-C6), of which the C1 subtype was characterized by extensive stromal desmoplasia and associated with the poorest survival [23]. Our analysis showed that SPHK1 mRNA was most highly expressed in the C1 subtype (Figure 2B). To further analyze SPHK1 in these molecular subtypes, we divided the AOCS dataset into SPHK1-High and SPHK1-Low groups by median transcript expression. Tumors that were classified as being of the C1 subtype constituted 54% of cases in the SPHK1-High group, but only 3.5% of cases in the SPHK1-Low group (Figure 2C). The opposite pattern was observed in the C4 subtype, which was defined by a low stromal response signature (Figure 2C). Similar analyses of the TCGA dataset demonstrated that high SPHK1 expression was associated with the mesenchymal subtype, which is thought to be equivalent to the C1 subtype and is associated with the poorest survival (Supplementary Figure S2A) [24].

Increased expression of α-smooth muscle actin (αSMA) and fibroblast activation protein (FAP) are frequently used to identify CAFs [22]. Transcript levels of *ACTA2* (the gene encoding αSMA) and FAP were significantly higher in SPHK1-High tumors than in SPHK1-Low tumors in both the AOCS and TCGA datasets (Figure 2D and Supplementary Figure S2B), suggesting that SPHK1 could be associated with an increased abundance of CAFs in ovarian tumors. Evaluation of SPHK1 expression in different laser-microdissected ovarian tissue components (GSE40595) showed that CAFs expressed significantly higher levels of SPHK1 than ovarian cancer cells (p=0.0002) or normal ovarian surface epithelium (OSE, p=0.0003) (Figure 2E and Supplementary Figure S3). We observed similar results in a panel of ovarian cancer cell lines, normal ovarian fibroblasts, and patient-derived omental fibroblasts and CAFs (Supplementary Figure S4).

### Ovarian cancer cells stimulate SPHK1 expression and induce CAF-like features in fibroblasts via TGF-β signaling

**Figure 3:**
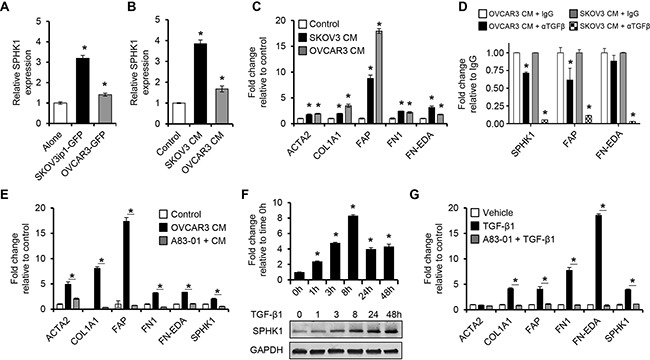
Ovarian cancer cells stimulate SPHK1 expression in ovarian fibroblasts via TGF-β1 signaling **A, B.** qRT-PCR analysis of SPHK1 mRNA expression in TRS3 ovarian fibroblasts cells **A.** cultured alone or cocultured with fluorescently labeled ovarian cancer cells for 48 hours, followed by FACS or **B.** stimulated with non-conditioned media (control) or conditioned media from ovarian cancer cells for 48 hours. **C.** Transcript levels of CAF markers in TRS3 cells incubated in ovarian cancer cell conditioned media for 48 hours. The mRNA level of each gene is indicated relative to its level in TRS3 cells incubated in non-conditioned media. **D.** Transcript levels of CAF markers in TRS3 cells incubated in ovarian cancer cell conditioned media for 6 hours with either anti-TGF-β antibody (α TGF-β) or IgG control. **E.** Induction of CAF markers by ovarian cancer conditioned media in TRS3 cells with or without pretreatment with the TGF-β type I receptor inhibitor A83-01. The mRNA level of each gene is expressed relative to its level in vehicle treated TRS3 cells (control). **F.** TRS3 cells were treated with TGF-β1 for the indicated times and mRNA (upper panel) and protein (lower panel) were harvested and evaluated for SPHK1 expression. **G.** Transcript levels of CAF-associated genes in TRS3 cells stimulated with TGF-β1 for 48 hours with or without pretreatment with A83-01. The mRNA level of each gene is expressed relative to its level in vehicle treated TRS3 cells. Data are presented as the mean ± SEM of at least three independent experiments. *p < 0.05. CM, conditioned media.

To investigate if SPHK1 expression in ovarian fibroblasts is induced by epithelial-stromal interaction, we cocultured immortalized human normal ovarian fibroblast cell lines (TRS3 and INOF) with fluorescently labeled ovarian cancer cells for 48 hours, and then separated by FACS. The cocultured ovarian cancer cell lines significantly induced SPHK1 mRNA in the fibroblasts in comparison to fibroblasts cultured alone (*p* < 0.05, Figure 3A and Supplementary Figure S5A). Similar results were obtained with conditioned medium derived from ovarian cancer cell lines (Figure 3B and Supplementary Figure S5B), suggesting that mediators secreted by ovarian cancer cells induce SPHK1 expression in ovarian fibroblasts. No significant changes in SPHK2 mRNA expression were observed (Supplementary Figure S6A-B). TRS3 and INOF cells differentiated into CAF-like cells when cultured with ovarian cancer cell conditioned medium as evidenced by the induced expression of the CAF markers ACTA2, FAP, collagen 1α (COL1A1), fibronectin (FN1), and fibronectin with an alternatively spliced domain A (FN-EDA) (*p* < 0.05, Figure 3C and Supplementary Figure S5C).

Studies have shown that ovarian cancer cells are an abundant source of TGF-β, a potent inducer of fibroblast activation in cancer [8, 22, 25, 26]. To confirm that ovarian cancer cells produce TGF-β, OVCAR3 and SKOV3 conditioned media were subjected to a TGF-β ELISA. Both cell lines secreted detectable amounts of TGF-β (Supplementary Figure S5D). To determine whether induction of SPHK1 expression was mediated by TGF-β produced by ovarian cancer cells, we stimulated TRS3 cells with ovarian cancer cell conditioned medium treated with an anti-TGF-β antibody or an IgG control. Anti-TGF-β treatment significantly inhibited CAF activation relative to IgG control treated conditioned media (Figure 3D). To elucidate whether CAF activation is dependent upon canonical TGF-β signaling via TGF-β receptors (TGFBRs) and SMAD3, we pretreated the fibroblast cell lines with either the TGF-β type I receptor inhibitors, A83-01 or ALK5 inhibitor II’ or the SMAD3 inhibitor, SIS3, before stimulating the cells with ovarian cancer cell conditioned medium. TRS3 cells pretreated with A83-01, ALK5 inhibitor II, and SIS3 had significantly lower expression of SPHK1 and CAF markers than the vehicle treated control (*p* < 0.05, Figure 3E and Supplementary Figure S5E). This effect was also seen with A83-01 pre-treatment of INOF cells (Supplementary Figure S5C). TGF-β1 stimulation of TRS3 and INOF cells increased SPHK1 mRNA expression by greater than two-fold (*p* < 0.001, Figure 3F and Supplementary Figure S5F), whereas no increase in SPHK2 mRNA was observed (Supplementary Figure S6C). TGF-β1 also significantly increased SPHK1 protein expression in a time-dependent manner (Figure 3F and Supplementary Figure S5F). To confirm that sphingosine kinase activity was also increased, S1P levels were measured by ELISA after TGF-β1 treatment. TGF-β1 treated TRS3 and INOF cells produced S1P at least four-fold greater than untreated cells (Supplementary Figure S5G). In addition, TGF-β1 by itself was able induce a CAF-like phenotype in TRS3 and INOF cells (Figure 3G and Supplementary Figure S5H), but this effect was abrogated by pretreatment of the ovarian fibroblasts with A83-01 (Figure 3F and Supplementary Figure S5H).

### Overexpression of SPHK1 in fibroblasts enhances myofibroblast differentiation and CAF-like function

**Figure 4:**
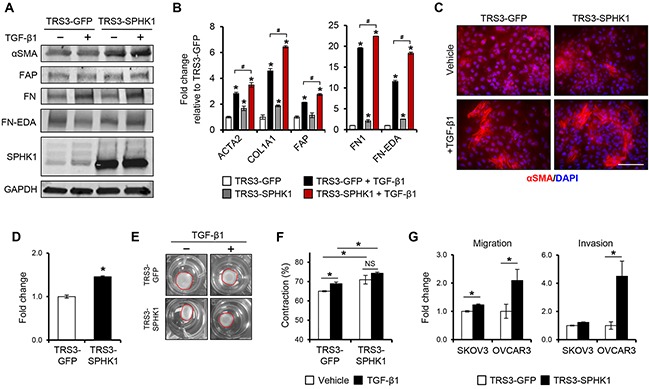
Overexpression of SPHK1 in ovarian fibroblasts enhances TGF-β1-induced myofibroblast marker expression and their ability to promote tumor cell migration and invasion **A.** Western blot and **B.** qRT-PCR analysis of CAF markers in TRS3-GFP and TRS3-SPHK1 cells 48 hours after stimulation with TGF-β1. The mRNA level of each gene is expressed relative to its level in vehicle treated TRS3-GFP cells. *p < 0.05 related to vehicle treated control; ^#^*p* < 0.05 related to TGF-β1-stimulated expression values as indicated. **C.** Immunofluorescence staining of αSMA in TRS3-GFP and TRS3-SPHK1 cells with and without TGF-β1 stimulation. Scale bar, 100 μm. **D.** Transwell migration of TRS3-GFP and TRS3-SPHK1 cells. Data are shown as fold change normalized to TRS3-GFP cells. **E.** Representative images of collagen gel contraction by TRS3-GFP and TRS3-SPHK1 cells at 24 hours. **F.** Quantification of collagen gel contraction. Data are presented as mean percent contraction compared to the total area of the well. **G.** SKOV3 or OVCAR3 cells were cocultured with TRS3-GFP or TRS3-SPHK1 cells for 48 hours. Migration and invasion of isolated ovarian cancer cells was assessed by transwell and matrigel invasion assay, respectively. Data are expressed as fold change normalized to ovarian cancer cells cocultured with TRS3-GFP cells. All data are presented as the mean ± SEM of at least three independent experiments. *p < 0.05. NS, not significant.

Given that SPHK1 is induced during myofibroblast differentiation and enriched in CAFs, we sought to determine if overexpression of SPHK1 could enhance fibroblast to myofibroblast conversion and promote CAF-like function. We generated TRS3 cell lines stably overexpressing SPHK1 (TRS3-SPHK1) or GFP (TRS3-GFP) (Figure 4A). TRS3-SPHK1 cells secreted increased levels of S1P relative to TRS3-GFP cells under normal growth conditions and after TGF-β1 treatment (Supplementary Figure S5G). Overexpression of SPHK1 in ovarian fibroblasts resulted in elevated basal and TGF-β1-induced protein and mRNA expression of the CAF markers αSMA, FAP, COL1A1, FN1, and FN-EDA (Figure 4A–4B). Immunofluorescence staining confirmed increased αSMA expression in TRS3-SPHK1 cells at both the basal level and after TGF-β1 stimulation (Figure 4C and quantified in Supplementary Figure S7A).

Myofibroblasts and CAFs are also characterized by an increased motility and ECM contraction [6, 27]. Indeed, TRS3-SPHK1 cells displayed increased migration (Figure 4D) and ∼5% increase in gel contraction over TRS3-GFP cells both with and without TGF-β1 stimulation (Figure 4E–4F). As CAFs are ultimately defined by their tumor promoting properties, we assessed the effect of SPHK1 overexpression in fibroblasts on the migration and invasion of ovarian cancer cells *in vitro*. Both the migration (∼2-fold) and invasion (∼4-fold) of OVCAR3 cells, and the migration (∼1.25-fold) of SKOV3 cells, were significantly enhanced by coculture with TRS3-SPHK1 cells in comparison to control TRS3-GFP cells (Figure 4G).

### SPHK1 and S1PR2/3 signaling are required for efficient myofibroblast differentiation and CAF-like function

**Figure 5:**
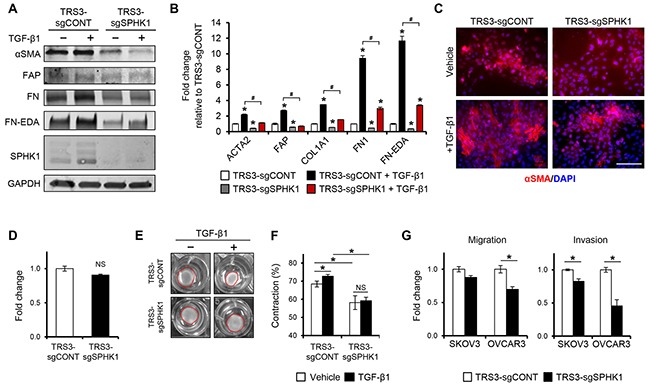
Knockout of SPHK1 in ovarian fibroblasts inhibits TGF-β1-induced myofibroblast differentiation and CAF-like function **A.** Western blot and **B.** qRT-PCR analysis of CAF markers in TRS3-sgCONT and TRS3-sgSPHK1 cells 48 hours after stimulation with TGF-β1. The mRNA level of each gene is expressed relative to its level in vehicle treated TRS3-sgCONT cells. *p < 0.05 related to vehicle treated control; ^#^*p* < 0.05 related to TGF-β1-stimulated expression values as indicated. **C.** Immunofluorescence staining of αSMA in TRS3-sgCONT and TRS3-sgSPHK1 cells with and without TGF-β1 stimulation. Scale bar, 100 μm. **D.** Transwell migration of TRS3-sgCONT and TRS3-sgSPHK1 cells. Data are shown as fold change normalized to TRS3-sgCONT cells. **E.** Representative images of collagen gel contraction by TRS3-sgCONT and TRS3-sgSPHK1 cells at 24 hours. **F.** Quantification of collagen gel contraction. Data are presented as mean percent contraction compared to the total area of the well. **G.** SKOV3 or OVCAR3 cells were cocultured with TRS3-sgCONT or TRS3-sgSPHK1 cells for 48 hours. Migration and invasion of isolated ovarian cancer cells was assessed by transwell and matrigel invasion assay, respectively. Data are expressed as fold change normalized to ovarian cancer cells cocultured with TRS3-sgCONT cells. All data are presented as the mean ± SEM of at least three independent experiments. *p < 0.05. NS, not significant.

**Figure 6:**
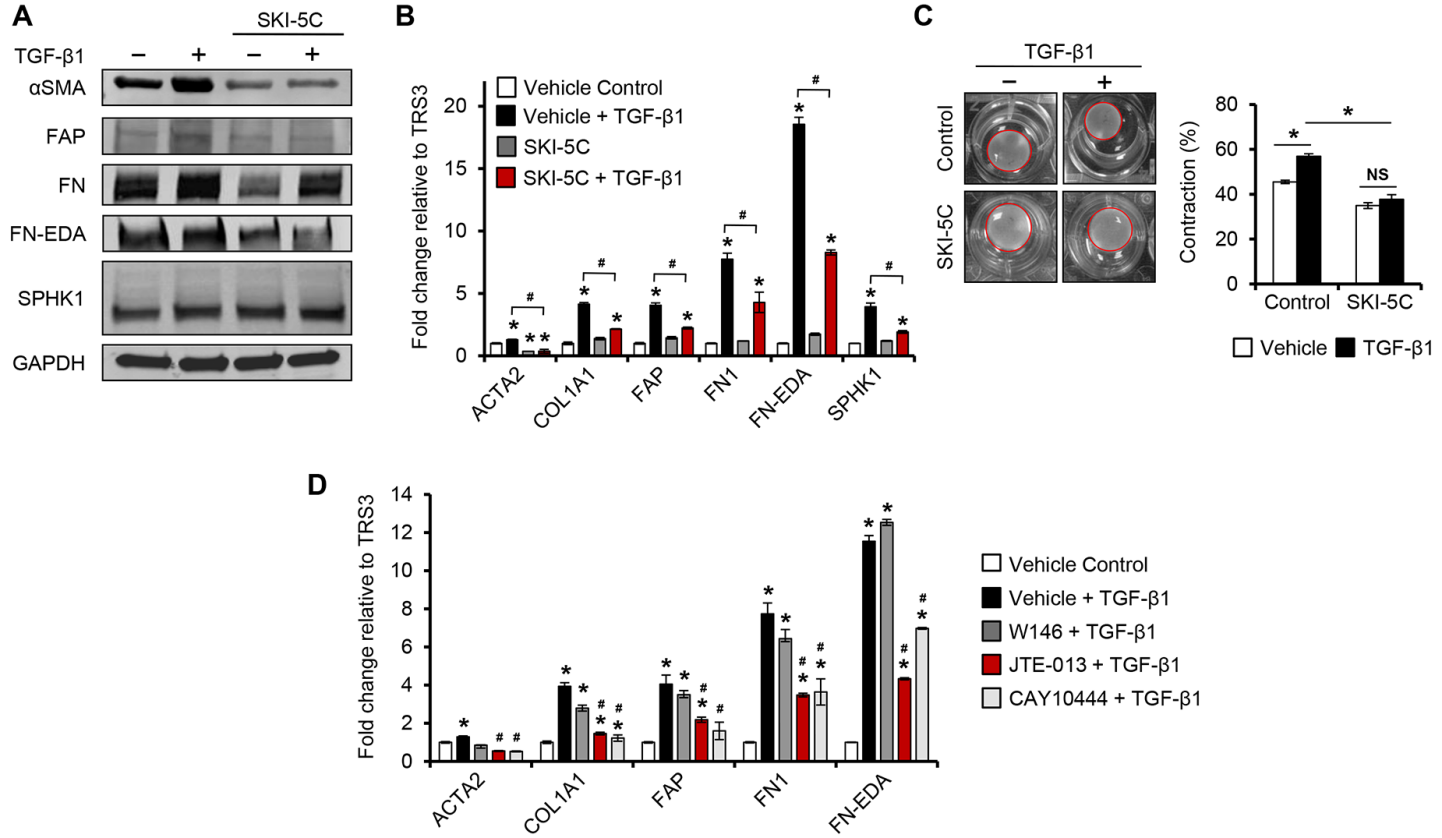
Pharmacological inhibition of SPHK1 or S1PR2/3 signaling inhibits TGF-β1-induced myofibroblast differentiation **A.** Western blot and **B.** qRT-PCR analysis of CAF markers in TRS3 cells pretreated with the SPHK1 inhibitor SKI-5C (5 μM) for 1 hour, and then stimulated with TGF-β1 for 48 hours. The mRNA level of each gene is expressed relative to its level in vehicle treated cells. *p < 0.05 related to vehicle treated control; ^#^*p* < 0.05 related to TGF-β1-stimulated expression values as indicated. **C.** Representative images of TGF-β-induced collagen gel contraction at 24 hours by TRS3 cells with and without pretreatment with SKI-5C. Data are presented as mean percent contraction compared to the total area of the well. **D.** TRS3 cells were incubated with S1PR1 antagonist W146 (10 μM), S1PR2 antagonist JTE-013 (10 μM), or S1PR3 antagonist CAY10444 (10 μM) for 1 hour before TGF-β1 stimulation for 48 hours. qRT-PCR analysis of CAF markers where the mRNA level of each gene is expressed relative to its level in vehicle treated cells. *p < 0.05 related to vehicle treated control; ^#^*p* < 0.05 related to TGF-β1-stimulated expression.

To determine if SPHK1 is required for TGF-β1-induced myofibroblast differentiation, we knocked out *SPHK1* in TRS3 cells (TRS3-sgSPHK1) via CRISPR-Cas9 (Figure 5A). *SPHK1* knockout cells secreted significantly less S1P in relative to control cells upon TGF-β1 treatment (Supplementary Figure S5G). The myofibroblastic phenotype was significantly attenuated in TRS3-sgSPHK1 cells and TGF-β1 was ineffective in inducing the expression of CAF markers (Figure 5A–5B). Immunofluorescence staining in TRS3-sgSPHK1 cells also confirmed a substantial decrease in the number of αSMA+ cells following TGF-β1 stimulation (Figure 5C and quantified in Supplementary Figure S7B). While migration ability of TRS3-sgSPHK1 cells did not change, their ability to contract collagen gels was decreased by ∼ 14% in comparison to control cells (Figure 5D–5F). Knockout of *SPHK1* also altered the ability of TRS3 cells to promote the migration and invasion of ovarian cancer cells *in vitro* (Figure 5G). Similar deficiencies in TGF-β1-induced myofibroblast differentiation and CAF-like function were observed following *SPHK1* knockout in the INOF ovarian fibroblast cell line (Supplementary Figure S8). To address whether pharmacological inhibition of SPHK1 activity could reproduce *SPHK1* knockout, we utilized SKI-5C, a selective inhibitor of SPHK1 enzymatic activity by binding to its active site [28]. Pharmacological inhibition of SPHK1 activity by SKI-5C attenuated TGF-β1-induced expression of CAF markers and decreased collagen gel contraction by ∼19% (Figure 6A–6C). Similar results were obtained in SKI-5C treated INOF cells (Supplementary Figure S9A-B).

It is known that activated SPHK1 translocates from the cytosol to the plasma membrane, leading to the formation of S1P, which is subsequently exported from the cell to act in both a paracrine and autocrine manner via cell surface S1P receptors (S1PRs) [11, 17]. Studies have shown that some of the downstream effects of TGF-β1 are also mediated by S1PR signaling [29–31]. To determine whether the myofibroblastic differentiation of ovarian fibroblasts is S1PR-mediated, we evaluated the effect of S1PR inhibition on the ability of TGF-β1 to induce CAF-associated gene expression. In TRS3 and INOF cells, qRT-PCR analysis showed mRNA expression of S1PR1, S1PR2, and S1PR3, while S1PR4 and S1PR5 were not detected (Supplementary Figure S10A). To ascertain which, if any, of these S1PRs were involved in mediating TGF-β1 signaling, TRS3 and INOF cells were stimulated with TGF-β1 in the presence of W146 (S1PR1 antagonist), JTE-013 (S1PR2 antagonist), or CAY10444 (S1PR3 antagonist). JTE-013 or CAY10444, but not W146, significantly reduced the induction of CAF markers by TGF-β1 (Figure 6D and Supplementary Figure S9C). We also found that TGF-β1 increases S1PR2 and S1PR3 mRNA (Supplementary Figure S10B), suggesting a feed-forward activation role for S1PR2 and S1PR3, but not S1PR1, in TGF-β1-induced myofibroblast differentiation.

### SPHK1 regulates TGF-β1-induced myofibroblast differentiation via S1PR2-mediated p38 MAPK activation

**Figure 7:**
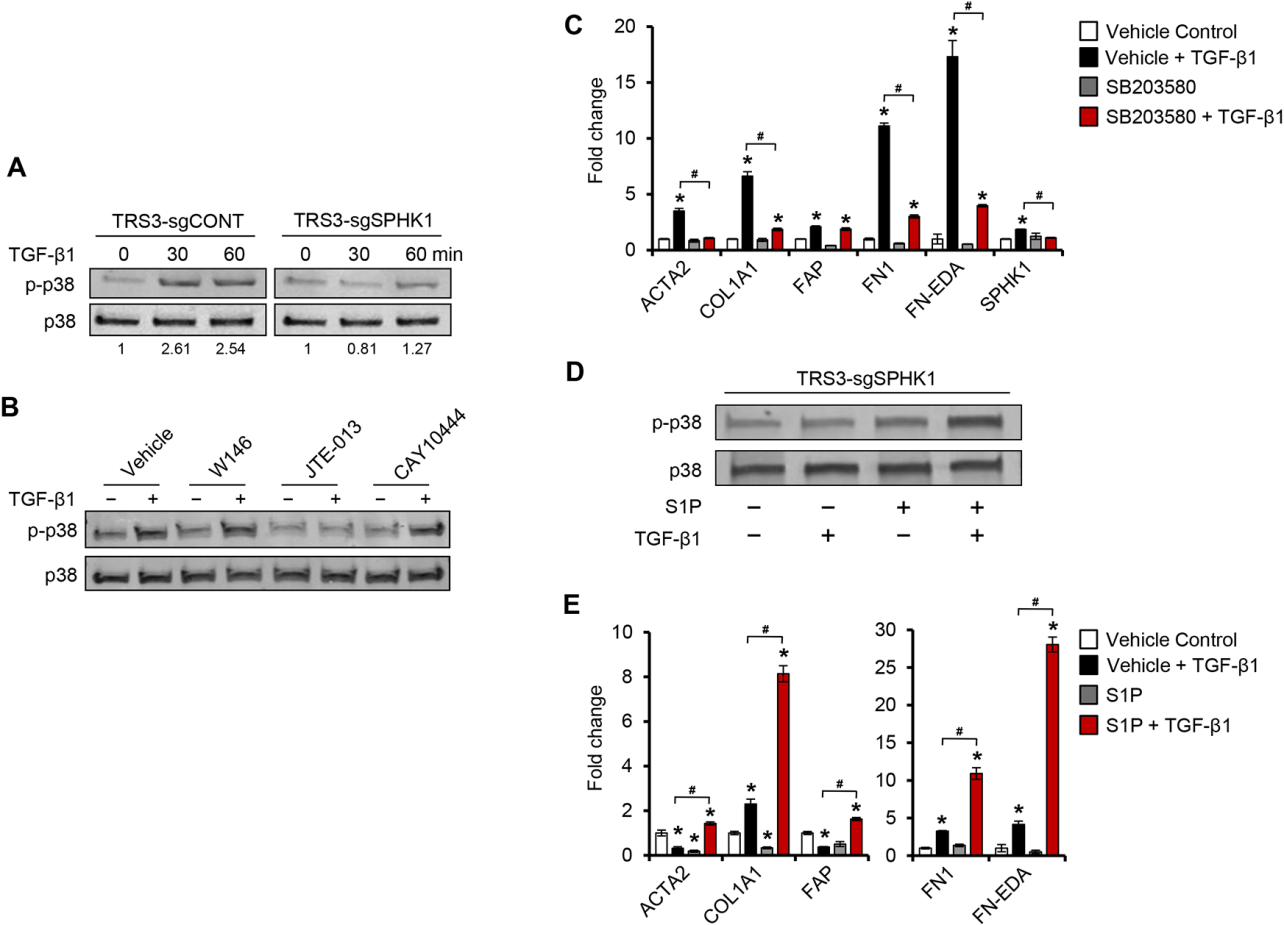
TGF-β1 induces p38 MAPK phosphorylation through S1PR2 and S1PR3 in ovarian fibroblasts **A.** Western blot analysis of phospho-p38 in TRS3-sgCONT and TRS3-sgSPHK1 after TGF-β1 stimulation for the indicated times. **B.** Western blot analysis of phospho-p38 in TRS3 cells pretreated with either S1PR1 antagonist W146 (10 μM), S1PR2 antagonist JTE-013 (10 μM), or S1PR3 antagonist CAY10444 (10 μM) for 1 hour, and then stimulated with TGF-β1 for 30 minutes. **C.** qRT-PCR analysis of CAF markers and ECM-associated genes in TRS3 cells pretreated with the p38 MAPK inhibitor SB203580 (10 μM) for 1 hour, and then stimulated with TGF-β1 for 48 hours. **D.** Western blot analysis of phospho-p38 in TRS3-sgSPHK1 cells pretreated with S1P (100 nM) for 1 hour before the addition of TGF-β1 for 30 minutes. **E.** qRT-PCR analysis of CAF markers in TRS3-sgSPHK1 cells pretreated with S1P (100 nM) for 1 hour, and then stimulated with TGF-β1 for 48 hours. For western blots, relative band intensities of phosphorylated proteins were quantified by densitometry, and the ratios of the phosphorylated to total signals are indicated below the blot. For each cell line the ratios are expressed as fold change from the untreated control. For qRT-PCR, the mRNA level of each gene is expressed relative to its level in vehicle treated cells. *p < 0.05 related to vehicle treated control; ^#^*p* < 0.05 related to TGF-β1-stimulated expression.

TGF-β signals through both Smad-dependent and –independent pathways. In renal mesangial cells and keratinocytes, the SPHK1/S1P axis has been shown to cross-activate and enhance Smad signaling [32, 33]. However in TRS3 and INOF cells, TGF-β1-induced Smad 2/3 phosphorylation was not significantly altered between control and SPHK1-knockout cells (data not shown), suggesting that effects of SPHK1 on ovarian myofibroblast differentiation are independent of Smad 2/3 signaling. Because S1PR2 and S1PR3 were required for efficient myofibroblast differentiation (Figure 6D), we investigated the effect of SPHK1 knockout on several of the signaling pathways that are downstream of these receptors, including ERK, Rac, and p38 MAPK [11, 34]. No change in the levels of activated phospho-ERK 1/2 or Rac1-GTP were detected (data not shown), however TRS3-sgSPHK1 cells had markedly reduced activation of p38 MAPK following TGF-β1 stimulation (Figure 7A). Reduced p38 MAPK activation by TGF-β1 was also observed in INOF-sgSPHK1 cells (Supplementary Figure S11A). To confirm p38 MAPK activation was indeed downstream of SPHK1 and S1PR2/3, we assessed the effect of S1PR inhibition on TGF-β1-induced p38 MAPK phosphorylation. Inhibition of S1PR2, but not S1PR1 or S1PR3, resulted in diminished levels of phosphorylated p38 MAPK (Figure 7B). Further, p38 MAPK activation was necessary for myofibroblast differentiation as pharmacological inhibition using SB203580 substantially inhibited the capacity of TGF-β1 to induce CAF-associated gene expression (Figure 7C and Supplementary Figure S11B). To examine the importance of S1P on TGF-β1-induced differentiation signals, we investigated whether exogenous S1P rescued the TGF-β1 effect in SPHK1-knockout cells. Exogenous S1P treatment rescued p38 MAPK phosphorylation (Figure 7D) and increased CAF-associated gene expression (Figure 7E and Supplementary Figure S11C) in SPHK1-knockout cells. Taken together, these data demonstrate that SPHK1, S1P, and intact S1PR2/3 signaling are essential for TGF-β1-induced myofibroblast differentiation through their regulation of p38 MAPK phosphorylation.

### Loss of stromal SPHK1 expression alters survival and tumor dissemination in a mouse model of ovarian cancer

**Figure 8:**
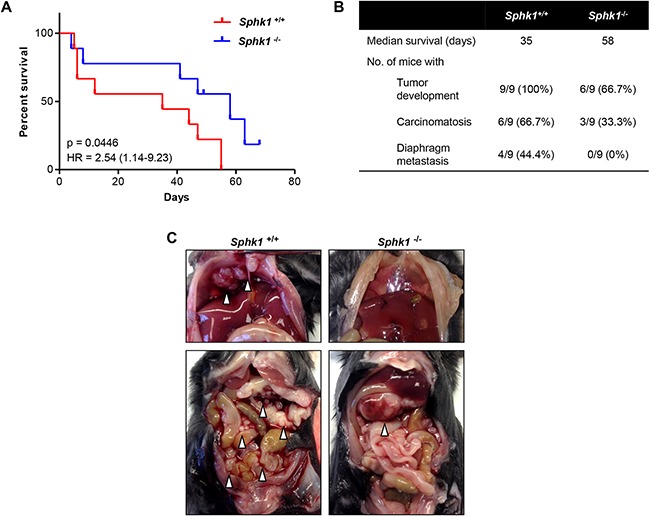
Stromal SPHK1 expression modulates tumor growth and dissemination in a mouse model of ovarian cancer **A.** Comparison of survival rates of female *Sphk1*^−/−^ and wild type (*Sphk1*^+/+^) mice after intraperitoneal injection of 5×10^6^ HRas-MOSE cells per mouse (*n* = 9 mice per group). **B.** Table summarizing the median survival time and number of mice per group that developed tumors, carcinomatosis, and diaphragm metastases. **C.** Representative photographs of tumor burden in female *Sphk1*^−/−^ and wild type mice. White arrowheads indicate tumor nodules.

To assess the effect of stromal SPHK1 expression on ovarian cancer growth and metastasis *in vivo*, a syngeneic mouse ovarian cancer cell line, MOSE-HRas (*p53*-null HRas-MOSE, Agadjanian *et al*. in preparation) was utilized. Similar to OVCAR3 and SKOV3, these cells secrete a significant amount of TGF-β and activate TRS3 fibroblasts in a TGF-β dependent manner (Supplementary Figures S5D and S12). MOSE-HRas cells were injected intraperitoneally into female SPHK1-knockout (*Sphk1*^−/−^) or wild type (*Sphk1*^+/+^) mice (*n* = 9 per group). *Sphk1*^−/−^ mice had significantly longer survival times than *Sphk1*^+/+^ mice (p = 0.0446) (Figure 8A–8B) and exhibited limited metastatic spread (Figure 8B–8C) suggesting that a microenvironment deficient in Sphk1 may be less proficient at promoting metastatic seeding. Overall, these results indicate that the growth and seeding of ovarian cancer cells are to some extent dependent on stroma-mediated paracrine signaling, and that SPHK1 expressed in the stroma may be a major player in this process.

## DISCUSSION

A majority of studies on SPHK1 have focused on its role in tumor cells. In HGSC cells, overexpression SPHK1 increased tumor cell proliferation, migration, and the development of chemoresistance while its knockdown or pharmacological inhibition resulted in reduced proliferation and enhanced apoptosis *in vitro* [11, 35]. Targeting of SPHK1 or S1P in pre-clinical mouse models of HGSC was also effective in inhibiting tumor growth [35, 36]. However, recent reports utilizing models of melanoma and colon cancer demonstrated that expression of SPHK1 and S1P by the tumor stroma might also be important in tumorigenesis [37, 38]. This study is an initial investigation into the potentially significant and unrecognized role of SPHK1 in the tumor stroma of HGSC. The importance of the tumor stroma has long been appreciated and it is now widely accepted that CAFs are pivotal in supporting tumor growth, progression, and invasion. Here, we show that endogenous fibroblast SPHK1 expression is required for TGF-β-induced myofibroblast differentiation and the acquisition of a CAF-like phenotype. The expression of SPHK1 is likely to be clinically relevant, as we report that SPHK1 is enriched in CAFs, and associated with a molecular subtype of HGSC characterized by a reactive stroma signature and poor prognosis.

Tumor cells secrete a variety of factors that induce changes in the surrounding stroma. Multiple studies in HGSC have demonstrated TGF-β to be the main driver of fibroblast activation and subsequent CAF formation [39–41]. Similarly, our data indicate that induction of a CAF-like phenotype in fibroblasts by ovarian cancer cells is mediated by TGF-β. Moreover, we show that SPHK1 is coordinately induced by TGF-β1 during this process. This finding is consistent with studies in models of organ fibrosis where SPHK1 was shown to be induced by TGF-β1 during myofibroblast differentiation [21, 29]. To determine if SPHK1 was required for TGF-β1-mediated CAF activation, we knocked out SPHK1 in ovarian fibroblasts. Loss of SPHK1 in fibroblasts abrogated the induction of myofibroblast markers αSMA and FAP, and markedly diminished the ability of fibroblasts to contract collagen gels or induce ovarian cancer cell migration and invasion. These findings are similar to those in studies in organ fibrosis, where SPHK1 was also shown to be required for myofibroblast activation and the secretion of ECM proteins [21, 29, 30]. However, we are the first to demonstrate that stromal SPHK1 expression is required for the myofibroblastic conversion and tumor-promoting effects of CAFs.

Previous studies have found SPHK1, S1P, and autocrine S1PR signaling to be crucial in facilitating some of TGF-β's downstream effects in myofibroblast differentiation [29, 30, 42]. These studies identified S1PR2 and/or S1PR3 as the key receptors in this process and determined they stimulated either ERK or Rho GTPase activity in a cell type specific manner (myoblasts versus lung fibroblasts) [29, 42]. Our data also confirmed the importance of S1PR2 in myofibroblastic differentiation of CAFs; however we did not find significant alterations in ERK or Rho GTPases. Instead we found knockout or inhibition of SPHK1 to result in reduced p38 MAPK phosphorylation. Although no studies have reported SPHK1 to activate p38 MAPK in fibroblasts, several studies have found p38 MAPK to be regulated by SPHK1 in other cell types [43–46]. In further support of our findings, p38 MAPK activation has been shown to be required for TGF-β-induced myofibroblast differentiation in dermal and tenon fibroblasts [47, 48]. A recent study also found p38 MAPK to be a key mediator of CAF function, specifically in the transcription and secretion of tumor-promoting growth factors and cytokines [49].

Effective targeting of CAFs requires the identification of factors and pathways that are necessary in facilitating their formation. In this study, we identified SPHK1, S1P, S1PR2, and p38 MAPK as essential for the TGF-β-mediated induction of myofibroblastic differentiation and the acquisition of a tumor-promoting CAF phenotype. Pharmacological inhibitors to each of these targets are readily available and some are already in clinical trials for cancer or other diseases [50, 51]. While further clinical investigation into the tolerability of these agents in patients is warranted, we believe that targeting SPHK1 and/or its downstream signaling machinery in HGSC may be advantageous for two reasons. First, whereas dysregulation of TGF-β signaling is recognized as the main driver of fibroblast-to-myofibroblast conversion in cancer, targeting this pathway is difficult due to its pleiotropic nature and carries the risk of adverse effects in patients; targeting a downstream mediator of TGF-β signaling such as SPHK1 should allow for more specific inhibition of the tumor-promoting aspects of TGF-β signaling [52, 53]. Second, SPHK1 inhibition would not only target the tumor stroma, but would also have direct effects on the tumor cells. Previous studies, as well as our own preliminary findings (data not shown), demonstrate that pharmacological inhibition of SPHK1 in HGSC cell lines induces cell death *in vitro* and reduces tumor growth *in vivo* [35]. While SPHK1 inhibition may improve patient outcome when combined chemotherapy, we hypothesize that SPHK1 inhibition may be even more useful as a consolidation or maintenance therapy by preventing the formation of the microenvironment required for the reemergence of HGSC. Using SPHK1 inhibitors in this manner would be similar to how the anti-VEGF antibody bevacizumab is currently used in some HGSC patients and has resulted in significant improvements in progression-free survival [54].

In summary, the current study suggests a novel role for SPHK1 in the activation and tumor-promoting role of CAFs. These data support a model in which ovarian cancer cells induce stromal SPHK1 expression via TGF-β, leading to inside-out S1P signaling through the S1PR2 receptor and the activation of p38 MAPK. Whereas targeting of TGF-β signaling is therapeutically challenging, inhibiting a downstream effector SPHK1 could be a promising therapeutic strategy aimed at the tumor stroma. This is significant for advanced stage HGSC as it is rarely cured by conventional chemotherapies.

## MATERIALS AND METHODS

### Human tissue specimens

Archived snap-frozen patient samples and paired clinical information were retrieved from the Women's Cancer Program Biorepository at Cedars-Sinai Medical Center. All patients were consented for biobanking, clinical data extraction, and molecular analysis. All cases were stage III/V serous epithelial ovarian cancer. This study was reviewed and approved by the Institutional Review Board at Cedars-Sinai Medical Center.

### Chemicals and reagents

Recombinant human TGF-β1 was purchased from Merck Millipore. TGF-β1 neutralizing antibody and control IgG antibody (Rabbit polyclonal IgG) were obtained from R&D systems. CAY10444, JTE-013, SB203580, sphingosine-1-phosphate (S1P), SKI-5C (CAY10621), SIS3, and W146 were obtained from Cayman Chemicals. A83-01 was purchased from Sigma-Aldrich. ALK5 inhibitor II was obtained from Enzo Life Sciences.

### Cell culture

The OVCAR3 cell line was obtained from Dennis Slamon (University of California, Los Angeles) in 2011. The SKOV3ip1-luc GFP cell line was provided by Ernst Lengyl (University of Chicago). All other human ovarian cancer cell lines were purchased from the American Type Culture Collection. Cell line authenticity was confirmed by Laragen using short tandem repeat (STR) method. The TRS3 cell line was derived from normal ovary and immortalized with SV40 T antigen [41]. The hTERT-immortalized normal ovarian fibroblast cell line INOF-tdTomato has been described [55]. All ovarian cancer cell lines were maintained in DMEM (Corning) supplemented with 10% fetal bovine serum (FBS), Plasmocin^™^ (2.5 μg/ml, Invivogen), and 1X Antibiotic-Antimycotic (GIBCO) unless otherwise indicated. The TRS3 and INOF cell lines were maintained in a 1:1 ratio of MCDB 105 (Sigma-Aldrich) and Medium 199 (GIBCO) with 10% or 15% FBS, respectively, and 1% penicillin-streptomycin. For conditioned media or TGF-β1 stimulation, 3×10^5^ TRS3 or INOF cells were seeded in 6-well plates, serum starved overnight, and then stimulated for the indicated time. Unless otherwise noted, cells were treated with 10 ng/mL of TGF-β1. Cells were pretreated with inhibitors for 1 hour before TGF-β1 stimulation. Generation of conditioned media is described in Supplementary Methods.

### RNA isolation and qRT-PCR analyses

For OpenArray Real-Time PCR, total RNA (2μg) was extracted from snap-frozen tumors using TRI Reagent (Molecular Research Center, Inc.) and reverse transcribed using the High-Capacity cDNA Reverse Transcription Kit (Applied Biosystems). cDNA was mixed with TaqMan OpenArray Real-Time Mix (Applied Biosystems) and loaded onto OpenArray Real-Time PCR plates containing the probes for *SPHK1* (Hs00184211_m1), SPHK2 (Hs01016543_g1), and internal control large ribosomal protein P0 (*RPLP0*, Hs99999902_m1). The qRT-PCR reactions were performed by the Cedars-Sinai Medical Center Genomics Core using the BioTrove OpenArray NT Cycler System and data were analyzed using the 2^−ΔCT^ method. After coculture of GFP-labeled ovarian cancer cells with ovarian fibroblasts, cells were separated by FACS in PBS with 0.5% BSA and RNA was extracted. For all other qRT-PCR analyses, RNA extraction was performed using the RNeasy Mini Kit (Qiagen) and reverse transcribed to cDNA using the Quantitect Reverse Transcription Kit (Qiagen). For qRT-PCR, 50 ng of cDNA was mixed with the appropriate primers and the iQ SYBR-Green Supermix (BioRad), and run on the CFX96 Real-Time System (BioRad). Data were analyzed using the 2^−ΔCT^ method. All mRNA data were normalized to RPL32 expression. Primer sequences are listed in Supplementary Table S3.

### Plasmid constructs and lentiviral transduction

pReciever-Lv105-SPHK1 and pReciever-Lv105-EGFP (GeneCopoeia) were co-transfected with delta 8.9 packaging plasmid and pCMV-VSVG plasmid obtained from Robert Weinberg (Addgene 8454) into Lenti-X^™^-293T cells (Clonetch) using Lipofectamine 2000 (Invitrogen). Cell medium was changed to DMEM supplemented with 30% FBS following overnight incubation. After 48 hours, cell medium was harvested and filtered using a 0.45 μm filter syringe. The viral supernatant was used with Polybrene (8 μg/ml) to transduce TRS3 and INOF cells lines. Cells were infected for 48 hours, following which polyclonal populations of cells were selected with puromycin (5 μg/ml) for at least 72 hours.

### Clustered regularly interspaced short palindromic repeats (CRISPR)-mediated gene knockout

Single guide RNAs (sgRNAs) were designed using the CRISPR Design Tool maintained by the Zhang Lab at the Massachusetts Institute of Technology (http://crispr.mit.edu/) to target the third coding exon of human *SPHK1* (Supplementary Table S3). sgRNAs were cloned into the LentiCRISPR V1 plasmid (Zhang Lab, Addgene Plasmid 49535) as previously described [56]. Transfection and viral supernatant collection were performed as described above. TRS3 and INOF cells were infected for 48 hours, following which polyclonal populations of cells were maintained with puromycin (5 μg/ml). Insertion of indels was confirmed using the GeneArt® Genomic Cleavage Detection Kit (Invitrogen) per the manufacture's protocol. When necessary, single clones were isolated using limiting dilution.

### Western blot analyses and antibodies

Whole cell lysates were prepared using RIPA lysis buffer (Sigma-Aldrich) containing protease and phosphatase inhibitor cocktail (Roche). Protein concentrations of cell lysates were determined by the BCA protein assay (Pierce). 30-50 μg of lysate were loaded onto a 4-20% gradient polyacrylamide gel (Bio-Rad) and subjected to gel electrophoresis. Resolved proteins were transferred to a PVDF membrane (Millipore) using the Trans-Blot® Turbo^™^ transfer system (Bio-Rad) at 25V for 10 minutes. Membranes were then blocked in Odyssey Blocking Buffer (Li-Cor) or 5% non-fat dry milk in TBS-T for 1 hour and then incubated with the appropriate antibody overnight at 4°C. Antibodies included: αSMA (Abcam ab5694), FAP (Abcam ab53066), FN detecting total fibronectin (Santa Cruz Biotechnology sc-9068), FN [IST-9] detecting only FN-EDA (Abcam ab6328), GAPDH (Fitzgerald Industries 10R-G109a; Acton, MA), p38α MAPK (Cell Signaling Technology (CST) #9217), phospho-p38 MAPK (CST #9211), SPHK1 (CST #12071), and SPHK2 (Santa Cruz Biotechnology sc-22704). Following overnight incubation at 4°C, the membranes were washed with TBS-T and then incubated with the appropriate IRDye ® secondary antibody (Li-Cor) for 1 hour at room temperature. Membranes were washed with TBS-T and the membrane signal was subsequently analyzed by the Li-Cor Odyssey system.

### Immunofluorescence

Cells were grown on coverslips and fixed with 4% paraformaldehyde for 15 minutes, washed with PBS, and permeabilized with ice-cold 100% methanol for 10 minutes at −20°C. The coverslips were then blocked with 5% normal goat serum in PBS for 1 hour at room temperature. This was followed by incubation with primary antibody for 1 hour at 37°C or overnight at 4°C depending on the manufacturer's recommendation. Antibodies included αSMA (Abcam ab5694) and FN detecting total fibronectin (Santa Cruz Biotechnology sc-9068). After washing with PBS, cells were incubated with the appropriate fluorescently conjugated secondary antibody (1:1000 dilution; Invitrogen) for 1 hour at 37°C. The coverslips were mounted on slides using Vectashield mounting medium with DAPI (Vector Labs).

### Transwell migration and invasion assays

For fibroblast migration, 1×10^5^ cells/mL were seeded in serum-free media onto 24-well Transwell inserts with 8.0 μm PET membranes (Merck Millipore). Media containing 10% FBS or 0% FBS were added to the bottom well as a chemoattractant and negative control, respectively. Migrated cells at 6 hours were fixed and stained with the Diff-Quik stain set (Siemens Healthcare Diagnostics) and counted in four different fields under an Olympus BX43 upright microscope. For ovarian cancer cell migration and invasion, cancer cells were cocultured for 48 hours with TRS3 or INOF cells using 0.4 μm Transwell inserts. After priming, ovarian cancer cells were seeded at 1×10^5^ cells/mL in serum-free media onto 24-well Transwell inserts with 8 μm PET membranes or Biocoat Matrigel invasion chambers (BD Biosciences). Media containing 10% FBS was used as a chemoattractant. Migrated cells at 6 hours or invaded cells at 48 hours were fixed and stained with the Diff-Quik stain set and counted in four different fields. The experiments were performed in triplicate wells and each experiment was performed at least two or three times as indicated.

### Collagen gel contraction assay

Cell culture dishes were coated with 1% BSA and incubated for 1 hour at 37°C to inhibit the collagen gels from attaching to the dishes. Collagen gels were prepared by mixing cell suspensions in 2X MCD105:199 medium with a neutralized solution of rat tail collagen type I matrix (9 parts collagen to 1 part neutralization buffer; Advanced BioMatrix #5153-A). The cell-collagen concentrations were adjusted with sterile PBS to attain a final collagen concentration of 1.0 mg/mL and a final cell concentration of 3×10^5^ cells/mL. The cell-collagen suspension was then added to the pretreated culture dishes and allowed to polymerize for 1 hour at 37°C. Serum-free medium with or without TGF-β1 was added to the solidified collagen gels. Collagen gel contraction was monitored over a period of 24 hours. To obtain gel contraction values, the relative diameters of the well and gel were measured using ImageJ software, and the percentage of gel contraction was calculated using the formula: [(well diameter-gel diameter)/well diameter]×100.

### Animal studies

All animal procedures were performed in accordance with the NIH Guide for the Care and Use of Laboratory Animals and approved by the Institutional Animal Care and Use Committee (IACUC) of Cedars-Sinai Medical Center. Sphk1-deficient (Sphk1*−/−*; B6N.129S6-Sphk1^tm1RIp^/J) mice were obtained from Jackson Laboratory (stock number 019095).[57] Six week-old female SPHK1-deficient (*Sphk1*^−/−^) and littermate-matched wild type (*Sphk1*^+/+^) controls were intraperitoneally injected with 5×10^6^ *p53*-null-HRas-mouse ovarian surface epithelial (MOSE) cells (Agadjanian *et al*. in preparation) in 200 μL of serum-free media. For survival studies, mice were euthanized by CO_2_ asphyxiation followed by cervical dislocation when morbid ascites developed or according to predefined criteria in order to avoid animal suffering. Tumor tissue, if present, was harvested and processed for subsequent hemotoxylin and eosin and immunohistochemical staining.

### Statistical analyses

Statistical analysis was performed using GraphPad Prism (version 6.0; GraphPad Software). Statistically significant data in *in vitro* and *in vivo* assays were assessed by unpaired Student's *t*-test unless otherwise noted. Data are expressed as the mean ± SEM. Intergroup differences were considered statistically significant when *p* < 0.05. Bioinformatic analysis of gene expression data for the Kaplan-Meier survival curves and from the AOCS and TCGA studies is described in the Supplementary Methods.

## SUPPLEMENTARY METHODS FIGURES AND TABLES




