# Supplementary material for: Sphingosine kinase 1 is required for TGF-β mediated fibroblast-to-myofibroblast differentiation in ovarian cancer
Source: Oncotarget. 2015 Dec 21;7(4):4167–82. doi: 10.18632/oncotarget.6703 (PMC4826197; doi:10.18632/oncotarget.6703)
Supplement: Supplementary file 1 [file oncotarget-07-4167-s001.pdf]

SUPPLEMENTARY METHODS

Gene expression analysis for progression-free and overall survival

The Kaplan-Meier Plotter (http://kmplot.com/) was used to determine the association of SPHK1expression with patient survival in a cohort of 13 gene expression data sets (n = 1137) including: GSE14764, GSE15622, GSE18520, GSE19829, GSE23554, GSE26193, GSE26712, GSE27651, GSE30161, GSE3149, GSE51373, GSE9891, and the TCGA ovarian cancer dataset. Datasets are available for download from the National Center for Biotechnology Information Gene Expression Omnibus (GEO) database and the TCGA data portal site (https://tcga-data.nci.nih.gov/tcga/). The Affymetrix probe 219257_s_at was utilized for SPHK1 expression. Patients were limited to those with serous histology and the auto-calculated best cutoff was used to define high and low SPHK1 expression. The Kaplan-Meier method was used to estimate progression-free and overall survival, and intergroup differences were assessed by log-rank test.

Australian Ovarian Cancer Study (AOCS) and the Cancer Genome Atlas (TCGA) Data Analysis

Gene expression data from ovarian cancer patients in the AOCS [1] and TCGA [2] studies were analyzed using the R2 Genomic Analysis and Visualization Platform (http://r2.amc.nl). The molecular subtype specific expression of SPHK1 was analyzed in both datasets. The expression level of SPHK1 was correlated with the expression level of all the genes in each dataset. Genes with a Pearson correlation greater than or equal to 0.6 and a false discovery rate less than 5% were selected from each cohort (Supplementary Table S1). The list of correlated genes in each cohort was analyzed using R2 Gene Ontology Analysis at a false discovery rate of less than 5% (Figure 2A and Supplementary Table S2). For analysis of activated fibroblast marker expression (ACTA2, FAP) patients were stratified by median expression of SPHK1.

Isolation of fibroblasts and CAFs from human samples

The tumor and tissue specimen samples used in this study were obtained with informed consent from all subjects. Fibroblasts were isolated from primary human omentum obtained from patients with stage III/IV serous ovarian cancer. Normal omentum was defined as omentum that appeared grossly normal with no evidence of metastatic implants. Normal and cancer-associated fibroblasts were isolated using an adapted previously described protocol. [3] Briefly, fresh tissue was minced, suspended in a 1X solution of collagenase/hyaluronidase (Stem Cell Technologies), and incubated for 6–8 hours on an orbital shaker at 37°C. After disassociation, the cell suspension was strained, and resulting suspension was centrifuged. The cell pellet was resuspended in DMEM with 10% FBS and 1% penicillin-streptomycin.

Coculture assays

For direct coculture, 2 X 10^6 fibroblasts were mixed with 1 X 10^6 GFP-labeled ovarian cancer cells (SKOV3ip1-luc GFP or OVCAR3-GFP) and cultured in a 10 cm dish for 48 hours. Cells were subsequently separated by FACS, and RNA was isolated. For indirect coculture, equal numbers of fibroblasts and ovarian cancer cells were cultured using a 0.4 μm Transwell insert.

Stimulation of cells with conditioned media

To generate tumor-conditioned media, 2 X 10^6 ovarian cancer cells were seeded in 10 cm dishes and cultured in serum-free DMEM medium for 48 hours. Tumor conditioned media was filtered using 0.45 μM syringe filter and stored at -80° until needed.

TGF-β1 and S1P quantitation by ELISA

Conditioned media obtained from ovarian cancer cells were subjected to the Human/Mouse TGF-β1 ELISA Ready-SET-Go assay following the manufacturer's protocol (eBioscience).

S1P levels from fibroblast conditioned media were determined by the Sphingosine Kinase Activity Kit following the manufacturer's protocol (Echelon Biosciences).

Mouse Genotyping

Mouse genomic DNA was isolated from tail biopsies following overnight digestion at 55°C in 300 μL DirectPCR Lysis Reagent (Tail) (Viagen Biotech, Inc) with 1 mg/mL Proteinase K solution. Lysates were heat inactivated by incubation at 85°C for 45 minutes. B6N.129S6-Sphk1^{tm1Rlp}/J mice were genotyped with GoTaqHot Start Colorless Master Mix 2X (Promega) per the manufacturer's instruction. Genotyping primers are listed in Supplementary information Table 3. PCR conditions were as follows: 94°C for 2 min, 10x [94°C for 20 s, 65°C for 15 s at -0.5°C per cycle, 68°C for 10 s], 28x [94°C for 15 s, 60°C for 15 s, 72°C s], 72°C for 2 min. PCR products were run on 1.2% agarose gel with ethidium bromide and visualized with the UVP Bioimaging System.

SUPPLEMENTARY REFERENCES

1. Tothill RW, Tinker AV, George J, Brown R, Fox SB, Lade S, Johnson DS, Trivett MK, Etemadmoghadam D, Locandro B, Traficante N, Fereday S, Hung JA, Chiew YE, Haviv I, Gertig D, et al. Novel molecular subtypes of serous and endometrioid ovarian cancer linked to clinical outcome. Clinical cancer research : an official journal of the American Association for Cancer Research. 2008; 14:5198–5208.
2. Integrated genomic analyses of ovarian carcinoma. Nature. 2011; 474:609–615.
3. Kenny HA, Krausz T, Yamada SD and Lengyel E. Use of a novel 3D culture model to elucidate the role of mesothelial cells, fibroblasts and extra-cellular matrices on adhesion and invasion of ovarian cancer cells to the omentum. International journal of cancer Journal international du cancer. 2007; 121:1463–1472.
4. Bonome T, Levine DA, Shih J, Randonovich M, Pise-Masison CA, Bogomolniy F, Ozbun L, Brady J, Barrett JC, Boyd J and Birrer MJ. A gene signature predicting for survival in suboptimally debulked patients with ovarian cancer. Cancer Res. 2008; 68:5478–5486.

SUPPLEMENTARY FIGURES AND TABLES

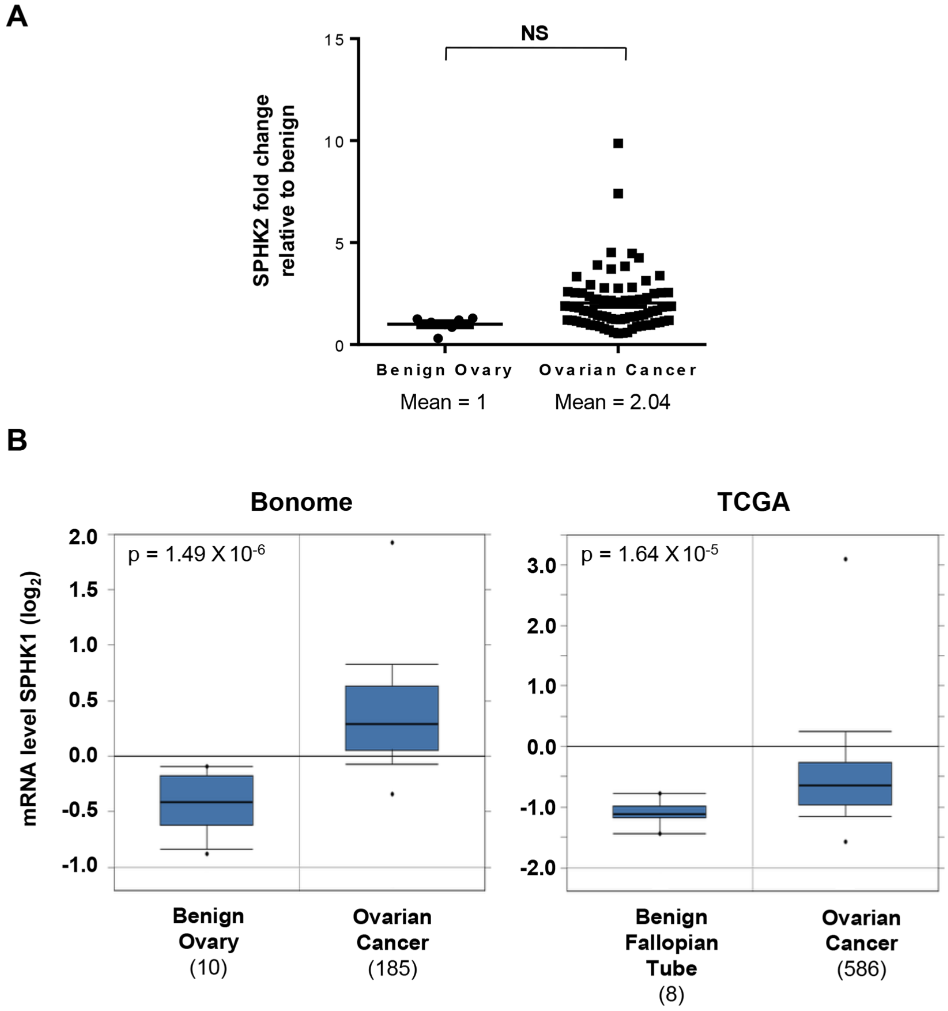

Supplementary Figure S1: SPHK1 but not SPHK2 is overexpressed in serous ovarian cancer. A. Quantification of SPHK2 mRNA in benign ovaries ($n = 7$) and ovarian cancer ($n = 77$) patient samples by OpenArray Real-Time PCR. Expression levels were normalized with RPLP0. Statistical significance was determined by Mann Whitney *U* test. **B.** The Oncomine expression analysis tool (www.oncomine.org) was used to examine SPHK1 expression in two large datasets; Bonome *et al*. [4] and TCGA. Statistical significance is based upon Student's *t*-test. The numbers in parentheses indicate the number of cases per group.

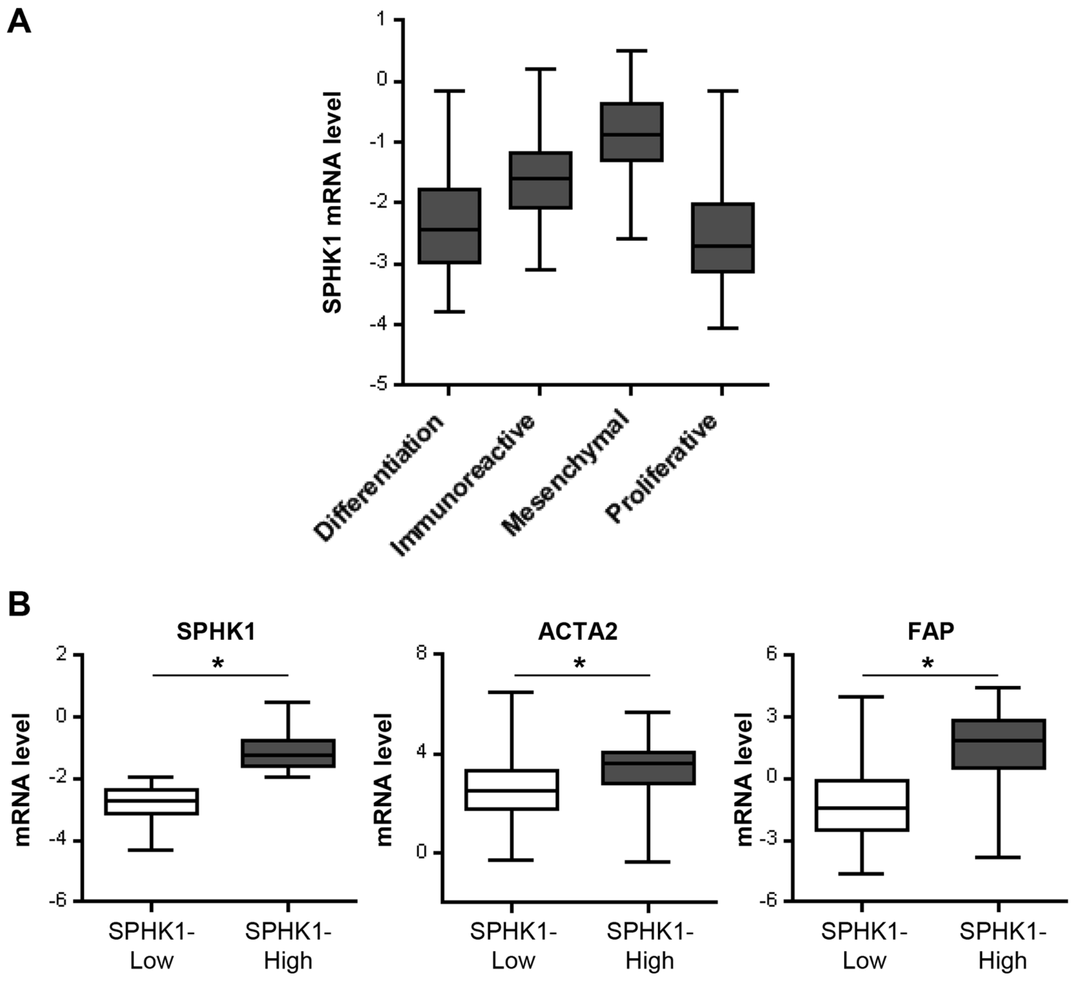

Supplementary Figure S2: SPHK1 expression is associated with the TCGA classified mesenchymal subtype. A. SPHK1 transcript levels associated with the molecular subtypes of high-grade serous ovarian cancer as defined by the TCGA [2]. The mesenchymal subtype was characterized by high expression of HOX genes and markers suggestive of increased stromal components such as myofibroblasts. **B.** Box-and-whisker plots of the differences in transcript levels of SPHK1, ACTA2 (encoding αSMA), and FAP between SPHK1-Low and SPHK1-High tumors. Median expression was used define SPHK1-Low and SPHK1-High samples. Statistical significance was determined by Mann Whitney *U* test. *$p < 0.05$

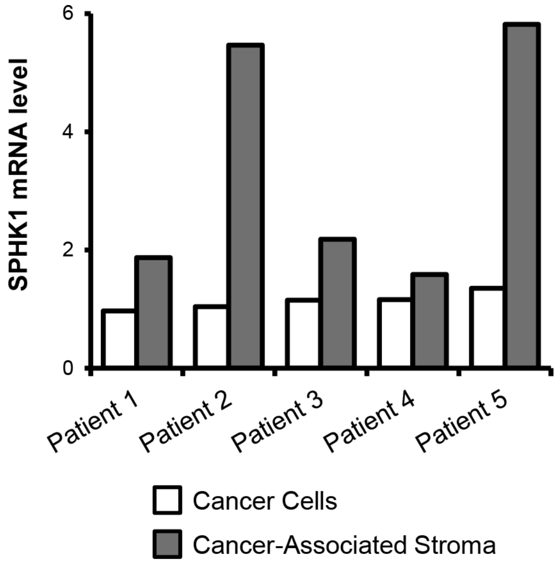

Supplementary Figure S3: SPHK1 expression in the AOCS dataset is associated with the stromal compartment. SPHK1 transcript levels in laser capture-microdissected cancer cell and stromal components of five patients with C1 subtype tumors from the AOCS dataset.

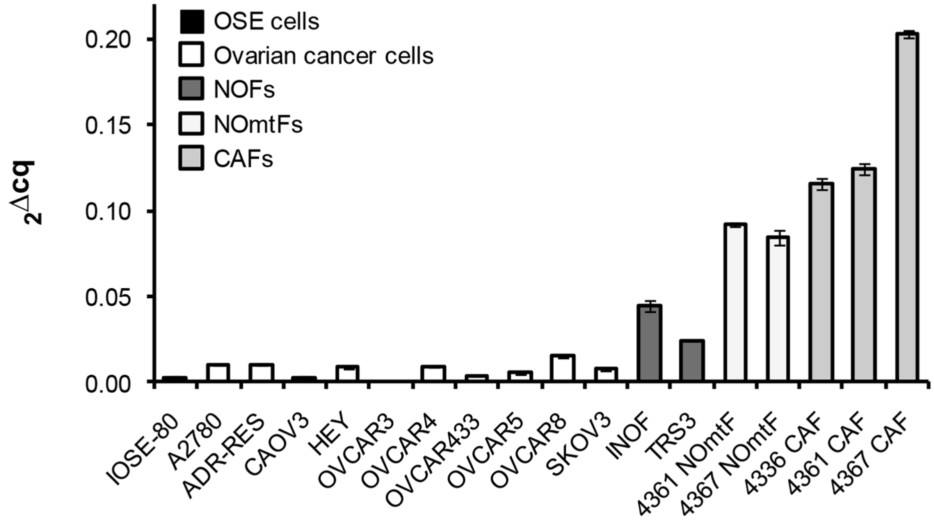

Supplementary Figure S4: Expression of SPHK1 mRNA in various ovarian cell lines. Expression levels of SPHK1 in immortalized ovarian surface epithelial (OSE) cells, ovarian cancer cells, immortalized normal ovarian fibroblasts (NOFs), patient-derived normal omental fibroblasts (NOmtFs), and patient-derived omental CAFs. Expression levels are normalized to *RPL32*. The data suggest that CAFs are the major source of SPHK1 expression in the ovarian tumor microenvironment.

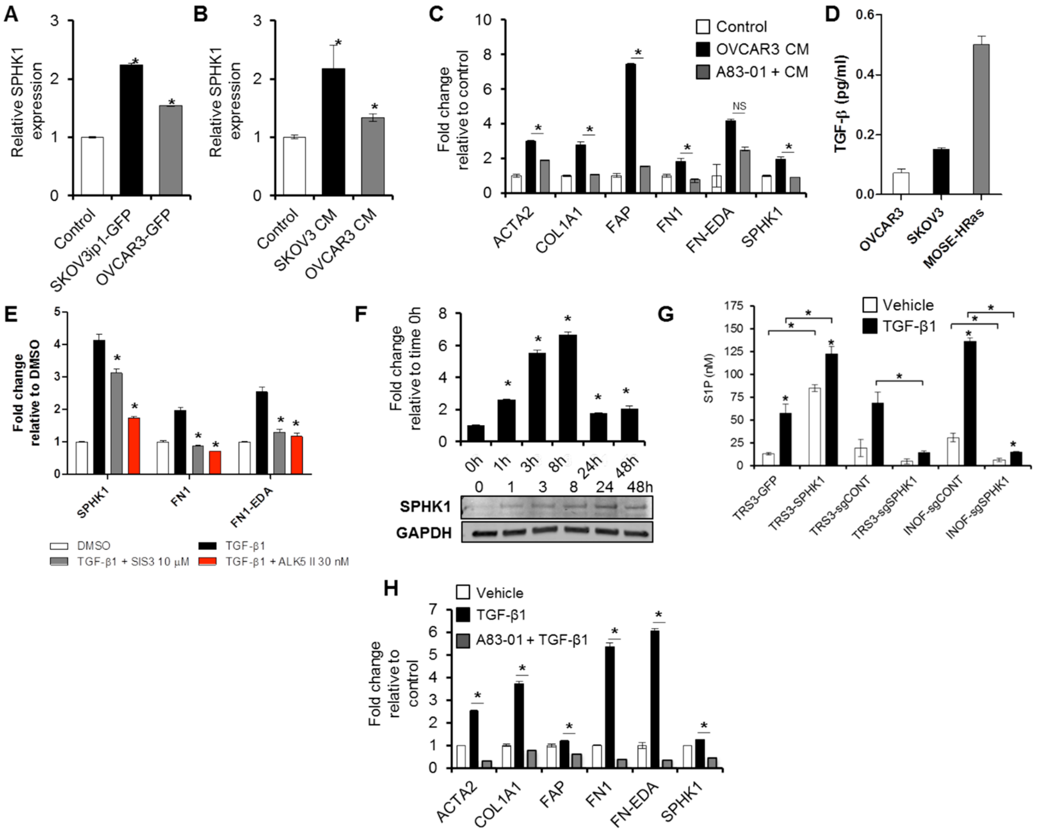

Supplementary Figure S5: Ovarian cancer cells and TGF-β1 stimulate SPHK1 expression and activity in INOF and TRS3 cells. **A. and B.** qRT-PCR analysis of *SPHK1* mRNA expression in INOF ovarian fibroblast cells **A.** cultured alone or cocultured with fluorescently labeled ovarian cancer cells for 48 hours, followed by FACS or **B.** stimulated with non-conditioned media or conditioned media from ovarian cancer cells for 48 hours. **C.** Induction of CAF-associated genes by ovarian cancer conditioned media in INOF cells with or without pretreatment with the TGF-β type I receptor inhibitor A83–01. The mRNA level of each gene is expressed relative to its level in untreated INOF cells. **D.** TGF-β ELISA of conditioned media from human and mouse ovarian cancer cell lines. **E.** Inhibition of TGF-β induced CAF activation in TRS3 cells by pre-treatment with ALK5 inhibitor II and SIS3. **F.** INOF cells were treated with TGF-β1 (10 ng/mL) for the indicated times and mRNA was harvested and evaluated for SPHK1 expression. **G.** Conditioned media from the indicated cell lines were harvested after 48 hours of treatment with TGF-β1 or vehicle control and extracellular S1P levels were detected by ELISA. **H.** Transcript levels of CAF-associated genes in INOF cells stimulated with TGF-β1 for 48 hours with or without pretreatment with A83–01. The mRNA level of each gene is expressed relative to its level in untreated INOF cells. Data are presented as the mean ± SEM of at least three independent experiments. $^{*}p < 0.05$. CM, conditioned media; NS, not significant

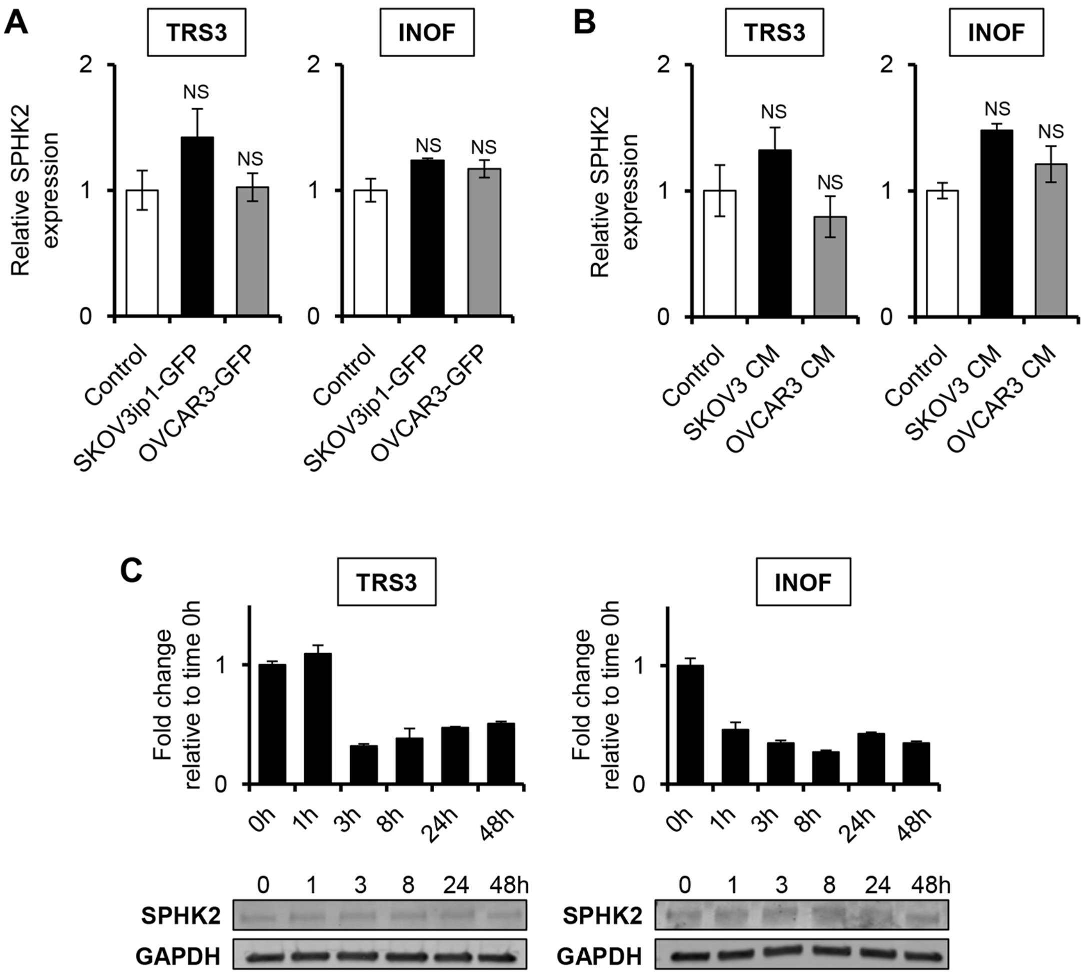

Supplementary Figure S6: SPHK2 is not induced by ovarian cancer cells or TGF-β1. A. and B. qRT-PCR analysis of SPHK2 mRNA expression in TRS3 and INOF cells (A) cocultured with fluorescently labeled ovarian cancer cells for 48 hours, followed by FACS, or (B) stimulated with conditioned media from ovarian cancer cells for 48 hours. **C.** TRS3 and INOF cells were treated with TGF-β1 (10 ng/mL) for the indicated times and mRNA (upper panel) and protein (lower panel) were harvested and evaluated for SPHK2 expression. GAPDH was used as a loading control. CM, conditioned media; NS, not significant

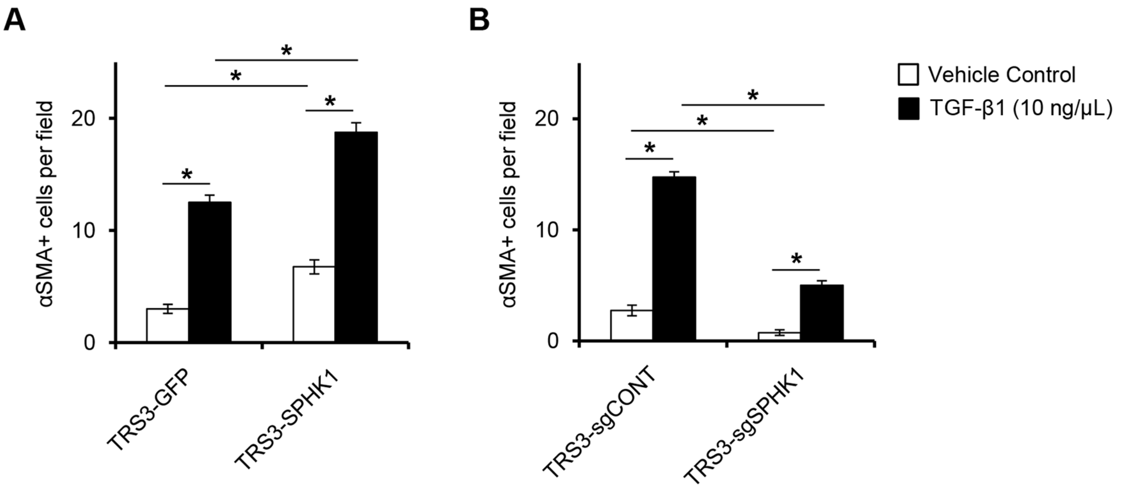

Supplementary Figure S7: Quantification of αSMA+ cells per field. A. TRS3-GFP and TRS3-SPHK1 or **B.** TRS3-sgCONT and TRS3-sgSPHK1 cells following TGF-β1 stimulation for 48 hours. *$p < 0.05$

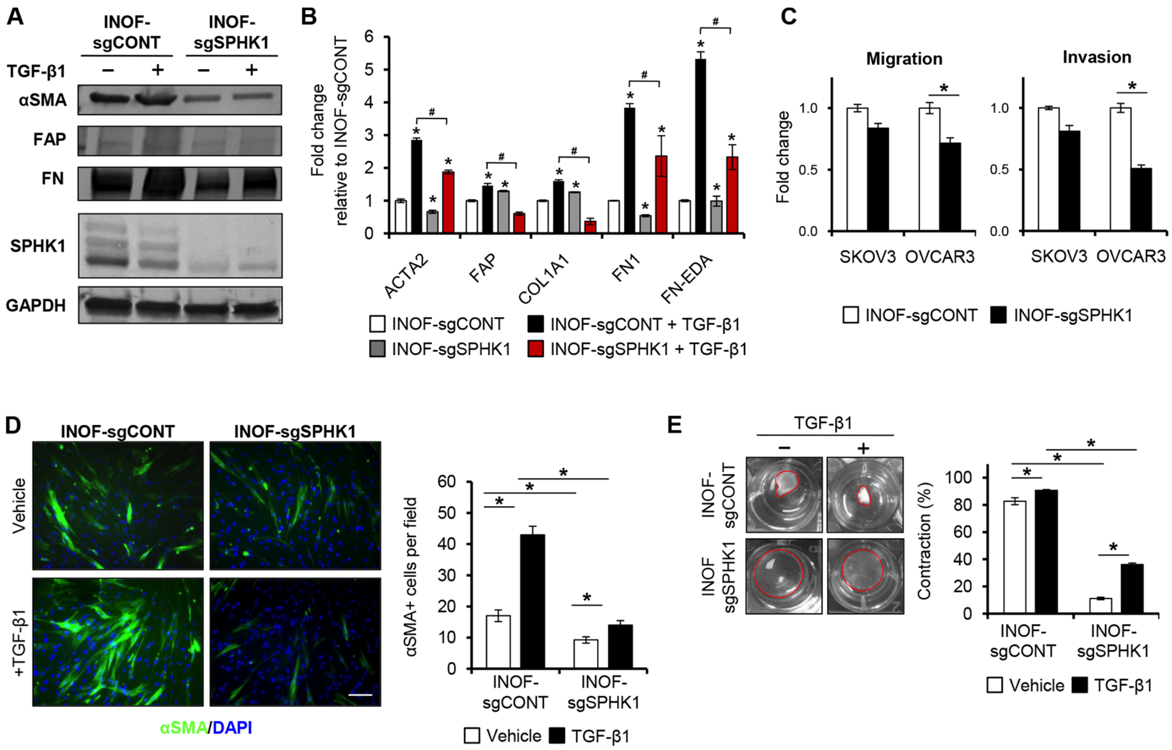

Supplementary Figure S8: Knockout of SPHK1 in INOF cells inhibits TGF-β1-induced myofibroblast differentiation and CAF-like function. A. Western blot and **B.** qRT-PCR analysis of CAF-associated makers in INOF-sgCONT and INOF-sgSPHK1 cells 48 hours after stimulation with TGF-β1. GAPDH was used as a loading control. The mRNA level of each gene is expressed relative to its level in vehicle treated control cells. *$p < 0.05$ related to vehicle treated control; $^{\#}p < 0.05$ related to TGF-β1-stimulated cell values as indicated. **C.** SKOV3 or OVCAR3 cells were cocultured with INOF-sgCONT or INOF-sgSPHK1 cells for 48 hours. Migration and invasion of isolated ovarian cancer cells was assessed by transwell and Matrigel invasion assay, respectively. Data are expressed as fold change normalized to ovarian cancer cells cocultured with INOF-sgCONT cells. **D.** Immunofluorescence staining of αSMA in INOF-sgCONT and INOF-sgSPHK1 cells with and without TGF-β1 stimulation. Scale bar, 100 μm. **E.** Representative images of collagen gel contraction by INOF-sgCONT and INOF-sgSPHK1 cells in 24 hours. Data are presented as mean percent contraction compared to the total area of the well. All data are presented as the mean ± SEM of at least three independent experiments. *$p < 0.05$.

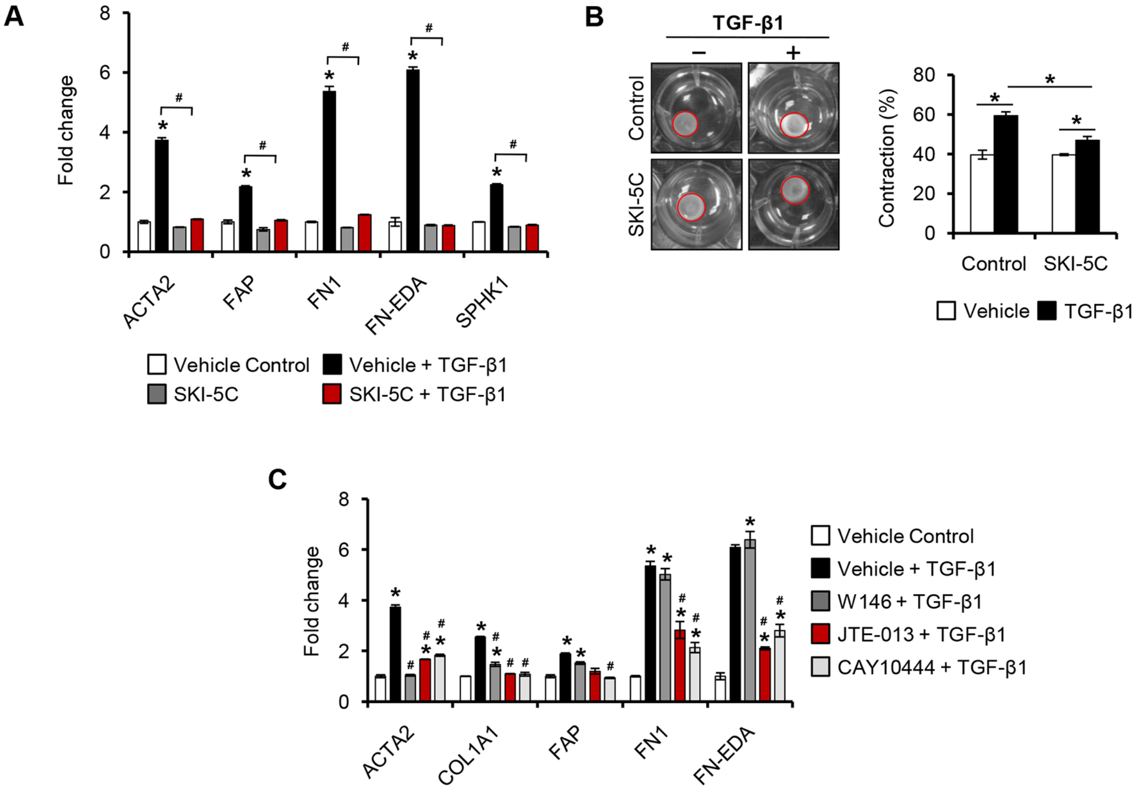

Supplementary Figure S9: Pharmacological inhibition of SPHK1 or S1PR2/3 receptor signaling inhibits TGF-β1-induced myofibroblast differentiation of INOF cells. A. qRT-PCR analysis of CAF-associated markers in INOF cells pretreated with the SPHK1 inhibitor SKI-5C (5 µM) for 1 hour, and then stimulated with TGF-β1 for 48 hours. GAPDH was used as a loading control. The mRNA level of each gene is expressed relative to its level in vehicle treated cells. $^{*}p < 0.05$ related to vehicle treated control; $^{\#}p < 0.05$ related toTGF-β1-stimulated expression values as indicated. **B.** Representative images of TGF-β-induced collagen gel contraction at 24 hours by TRS3 cells with and without pretreatment with SKI-5C. Data are presented as mean percent contraction compared to the total area of the well. **C.** TRS3 cells were incubated with S1PR1 inhibitor W146 (10 µM), S1PR2 inhibitor JTE-013 (10 µM), or S1PR3 inhibitor CAY10444 (10 µM) for 1 hour before TGF-β1 stimulation for 48 hours. qRT-PCR analysis of CAF-associated markers. The mRNA level of each gene is expressed relative to its level in vehicle treated TRS3 cells. $^{*}p < 0.05$ related to vehicle treated control; $^{\#}p < 0.05$ related to TGF-β1-stimulated expression.

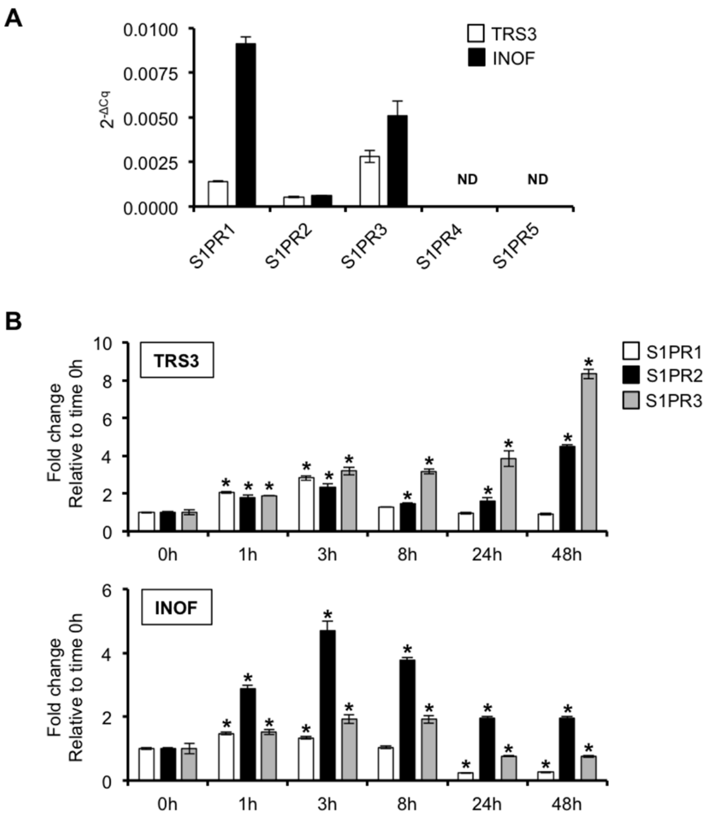

Supplementary Figure S10: The effect of TGF-β1 on the expression of S1P receptors (S1PRs) in ovarian fibroblasts. A. Basal mRNA expression levels of all five S1P receptors in TRS3 and INOF cell lines were analyzed by qRT-PCR. Expression levels are normalized to RPL32. ND, not detected. **B.** mRNA expression levels of S1PR1, S1PR2, and S1PR3 upon TGF-β1 treatment for the indicated amounts of time. * p-value < .01.

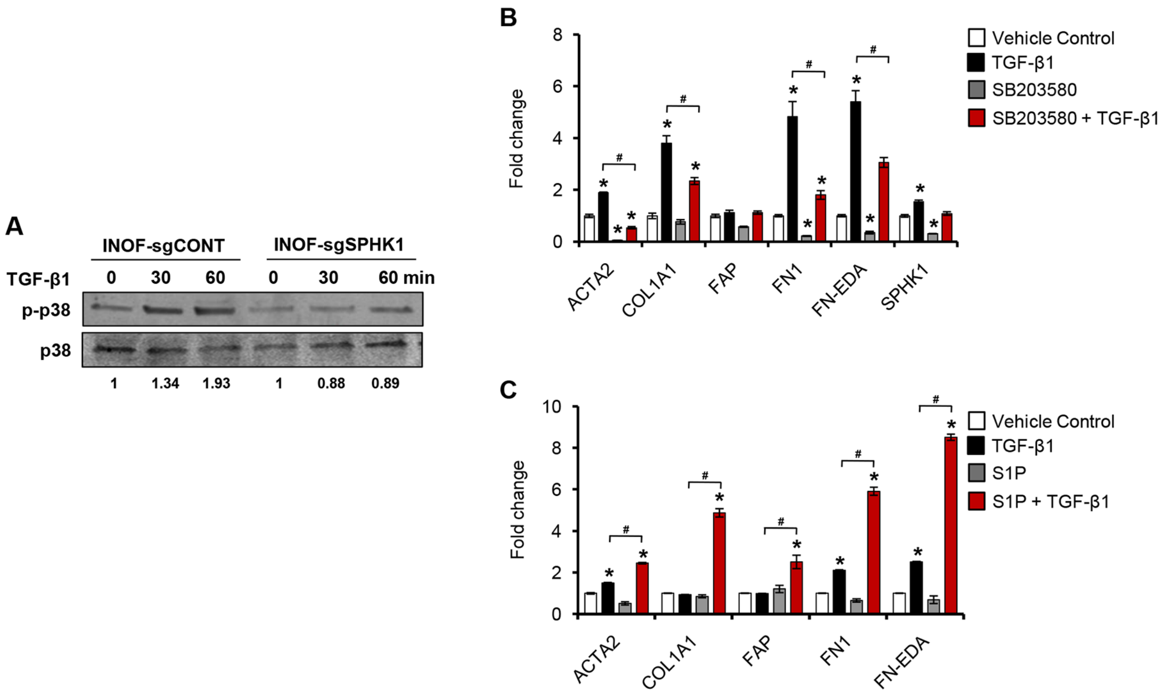

Supplementary Figure S11: TGF-β1 induces p38 MAPK phosphorylation through S1PR2 and S1PR3 in INOF cells. A. Western blot analysis of phospho-p38 in INOF-sgCONT and INOF-sgSPHK1 after TGF-β1 stimulation for the indicated times. **B.** qRT-PCR analysis of CAF-associated markers in INOF cells pretreated with the p38 MAPK inhibitor SB203580 (10 μM) for 1 hour, and then stimulated with TGF-β1 for 48 hours. **C.** qRT-PCR analysis of CAF-associated markers in INOF-sgSPHK1 cells pretreated with S1P (100 nM) for 1 hour, and then stimulated with TGF-β1 for 48 hours. For western blot, relative band intensities of phosphorylated p38 MAPK were quantified by densitometry, and the ratios of the phosphorylated to total signals are indicated below the blot. For each cell line the ratios are expressed as fold change from the untreated control. For qRT-PCR, the mRNA level of each gene is expressed relative to its level in vehicle treated cells. *$p < 0.05$ related to vehicle treated control; $^{\#}p < 0.05$ related to TGF-β1-stimulated expression.

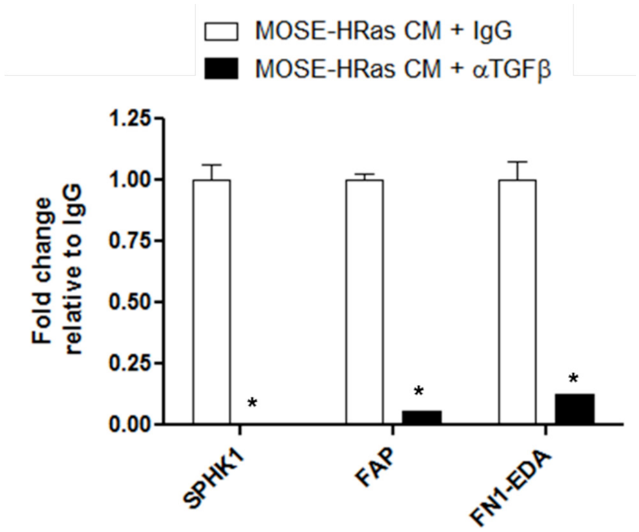

Supplementary Figure S12: MOSE-HRas cells induce SPHK1and CAF-activation markers in a TGF-β dependent manner. Conditioned media from MOSE-HRas cells were treated with either anti-TGF-β antibody (α TGFβ) or IgG control. qRT-PCR analysis was performed after incubation of TRS3 cells with the treated conditioned media for 6 hours. * p-value < .01.

Supplementary Table S1: Genes with expression levels significantly correlated (R >0.60) with that of SPHK1 in either the TCGA or the AOCS datasets (false discovery rate of <0.5%). Bolded genes are positively correlated in both datasets

See Supplementary File 1

Supplementary Table S2: Significant biological processes that correlate with *SPHK1* expression in the TCGA dataset (false discovery rate < 5%) identified using R2 Gene Ontology Analysis

**Positive Correlation (≥0.6) with SPHK1**		
**GO term**	***p*-value**	**Genes**
Proteinaceous extracellular matrix	1.19E-115	*POSTN, C1QTNF6, CTHRC1, COL1A1, COL1A2, COL5A1, COL5A2, COL6A2, COL6A3, COL8A1, COL11A1, COL6A6, VCAN, EPYC, ECM1, FBN1, FGF1, FN1, LAMA4, LGALS1, LOX, LOXL2, LUM, MMP2, MMP11, MMP13, MMP19, COL5A3, ASPN, CD248, TNN, SPARC, TGFBI, THBS2, COLEC12, ADAMTS4*
Collagen fibril organization	1.8E-102	*COL1A1, COL1A2, COL5A1, COL5A2, COL11A1, GREM1, LOX, LOXL2, LUM, COL5A3, SFRP2*
ECM organization and biogenesis	5.9E-65	*POSTN, COL1A1, COL1A2, COL5A1, COL5A2, COL6A2, COL6A3, COL8A1, COL11A1, FN1, OLFML2B, MMP2, MMP11, MMP13, MMP14, COL5A3, TGFBI, CRISPLD2*
Cell adhesion	3.9E-33	*POSTN, COL5A1, COL6A3, COL8A1, COL6A6, VCAN, AEBP1, ITGA11, FN1, ITGA5, LAMA4, LOXL2, CCL11, TGFBI, THBS1, THBS2, TNFAIP6, VCAM1, SVEP1, ADAM12, WISP1, ITGBL*
Positive regulation of cell migration	1.4E-24	*CCL26, PDPN, COL1A1, FGF1, MMP14, PDGFRB, CCL11, SNAI2, THBS1, SPHK1*
Metalloendopeptidase activity	1.4E-22	*FAP, MMP2, MMP11, MMP13, MMP14, MMP19, ADAM12, ADAM19, ADAMTS4*

Supplementary Table S3: Primer and Oligo Sequences

**qRT-PCR Primer Sequences (5′ to 3′)**			
ACTA2 F	CTATGCCTCTGGACGCACAACT	S1PR1 F	GCACCAACCCCATCATTTA
ACTA2 R	CAGATCCAGACGCATGATGGCA	S1PR1 R	TTGTCCCCTTCGTCTTTCTG
COL1A1 F	GAGGGCCAAGACGAAGACATC	S1PR2 F	CAAGTTCCACTCGGCAATGT0
COL1A1 R	CAGATCACGTCATCGCACAAC	S1PR2 R	CAGGAGGCTGAAGACAGAGGG
FAP F	GGAAGTGCCTGTTCCAGCAATG	S1PR3 F	TCAGGGAGGGCAGTATGTTC
FAP R	TGTCTGCCAGTCTTCCCTGAAG	S1PR3 R	GAGTAGAGGGGCAGGATGGT
FN1 F	GGCTGACAGAGAAGATTCCCGAGAG	S1PR4 F	AGCCTTCTGCCCCTCTACTC
FN1 R	CCAGTTTAGATGGATCTTGGCAGAGAGAC	S1PR4 R	ATCAGCACCGTCTTCAGCA
FN-EDA F	TAAAGGACTGGCATTCACTGA	S1PR5 F	ACAACTACACCGGCAAGCTC
FN-EDA R	GTGCAAGGCAACCACACTGAC	S1PR5 R	GCCCCGACAGTAGGATGTT
MMP2 F	GATACCCCTTTTGACGGTAAGGA	SPHK1 F	AAGACCTCCTGACCAACTGC
MMP2 R	CCTTCTCCCAAGGTCCATAGC	SPHK1 R	GGCTGAGCACAGAGAAGAGG
MMP9 F	CCCTGGAGACCTGAGAACCA	SPHK2 F	CTGTCTGCTCCGAGGACTGC
MMP9 R	CCACCCGAGTGTAACCATAGC	SPHK2 R	CAAAGGGATTGACCAATAGAAGC
RPL32 F	ACAAAGCACATGCTGCCCAGTG		
RPL32 R	TTCCACGATGGCTTTGCGGTTC		
**CRISPR Oligo Sequences (5′ to 3′)**			
sg1-SPHK1 F	CTGAACCCGCGCGGCGGCAA		
sg1-SPHK1 R	GCCTTGGGCGCGCCGCCGTT		
**CRISPR Cleavage Detection Primer Sequences (5′ to 3′)**			
SPHK1 F	AAGTTCCCACACTAGTGCCC		
SPHK1 R	CACCACCTGTTGAAGCACAC		
**SPHK1 Mouse Genotyping Primer Sequences (5′ to 3′)**			
Wildtype F	TCATCAACTGCACACTGCTG		
Mutant F	GCTATCAGGACATAGCGTTGG		
Common R	GTCCAATGAGAAGGCACTGG		
