# Supplementary material for: Sphingosine kinase 1 is required for TGF-β mediated fibroblast-to-myofibroblast differentiation in ovarian cancer
Source: Oncotarget. 2015 Dec 21;7(4):4167–82. doi: 10.18632/oncotarget.6703 (PMC4826197; doi:10.18632/oncotarget.6703)
Supplement: Supplementary file 2 [file oncotarget-07-4167-s002.docx]

**Supplementary Table 1** Genes with expression levels significantly correlated (R >0.60) with that of SPHK1 in either the TCGA or the AOCS datasets (false discovery rate of <0.5%). Bolded genes are positively correlated in both datasets.

|  |  | **Correlation Coefficient (R)** | |  |  |  | **Correlation Coefficient (R)** | |
| --- | --- | --- | --- | --- | --- | --- | --- | --- |
|  | **Gene Symbol** | **TCGA** | **AOCS** |  |  | **Gene Symbol** | **TCGA** | **AOCS** |
| 1 | SPHK1 | 1 | 1 |  | 34 | **ALPK2** | 0.677 | 0.623 |
| 2 | ECM1 | 0.782 | <0.6 |  | 35 | TDO2 | 0.676 | <0.6 |
| 3 | **FAP** | 0.775 | 0.642 |  | 36 | **COL5A1** | 0.676 | 0.639 |
| 4 | **ADAM12** | 0.77 | 0.643 |  | 37 | CD248 | 0.675 | <0.6 |
| 5 | **FN1** | 0.763 | 0.64 |  | 38 | CCL11 | 0.674 | <0.6 |
| 6 | **INHBA** | 0.762 | 0.656 |  | 39 | GPR68 | 0.673 | <0.6 |
| 7 | **TNFAIP6** | 0.761 | 0.642 |  | 40 | WISP1 | 0.672 | <0.6 |
| 8 | **THBS2** | 0.757 | 0.681 |  | 41 | IL7R | 0.671 | <0.6 |
| 9 | **ITGA5** | 0.757 | 0.622 |  | 42 | MMP2 | 0.668 | <0.6 |
| 10 | CTSK | 0.746 | <0.6 |  | 43 | GFPT2 | 0.664 | <0.6 |
| 11 | **PLAU** | 0.744 | 0.677 |  | 44 | PPEF1 | 0.662 | <0.6 |
| 12 | **COL5A2** | 0.741 | 0.651 |  | 45 | PDPN | 0.662 | <0.6 |
| 13 | **OLFML2B** | 0.731 | 0.701 |  | 46 | COL5A3 | 0.66 | <0.6 |
| 14 | **POSTN** | 0.731 | 0.645 |  | 47 | COL6A2 | 0.658 | <0.6 |
| 15 | **SERPINF1** | 0.728 | 0.63 |  | 48 | GJB2 | 0.655 | <0.6 |
| 16 | HNT | 0.727 | <0.6 |  | 49 | SFRP2 | 0.654 | <0.6 |
| 17 | VCAN | 0.719 | <0.6 |  | 50 | THBS1 | 0.652 | <0.6 |
| 18 | **GREM1** | 0.712 | 0.621 |  | 51 | **PRRX1** | 0.651 | 0.606 |
| 19 | **LUM** | 0.711 | 0.61 |  | 52 | ADAMTS4 | 0.648 | <0.6 |
| 20 | LRRC15 | 0.71 | <0.6 |  | 53 | **SPARC** | 0.648 | 0.607 |
| 21 | ITGA11 | 0.707 | <0.6 |  | 54 | FGF1 | 0.648 | <0.6 |
| 22 | PTGIR | 0.706 | <0.6 |  | 55 | SNAI2 | 0.646 | <0.6 |
| 23 | COPZ2 | 0.701 | <0.6 |  | 56 | HOM-TES | 0.644 | <0.6 |
| 24 | TGFBI | 0.699 | <0.6 |  | 57 | **CTHRC1** | 0.644 | 0.601 |
| 25 | LGALS1 | 0.696 | <0.6 |  | 58 | MSC TF | 0.643 | <0.6 |
| 26 | TMEM158 | 0.695 | <0.6 |  | 59 | VCAM1 | 0.641 | <0.6 |
| 27 | COL11A1 | 0.694 | <0.6 |  | 60 | PDGFRB | 0.641 | <0.6 |
| 28 | BHLHB5 | 0.693 | <0.6 |  | 61 | **AEBP1** | 0.641 | 0.619 |
| 29 | IGFL2 | 0.691 | <0.6 |  | 62 | FBN1 | 0.638 | <0.6 |
| 30 | **MMP11** | 0.688 | 0.629 |  | 63 | COL1A1 | 0.638 | <0.6 |
| 31 | ANGPTL2 | 0.682 | <0.6 |  | 64 | COLEC12 | 0.637 | <0.6 |
| 32 | LOXL2 | 0.68 | <0.6 |  | 65 | EPYC | 0.636 | <0.6 |
| 33 | **RAB31** | 0.678 | 0.618 |  | 66 | MMP13 | 0.635 | <0.6 |
|  |  | **Correlation Coefficient (R)** | |  |  |  |  |  |
|  | **Gene Symbol** | **TCGA** | **Tothill** |  |  |  |  |  |
| 67 | MMP19 | 0.634 | <0.6 |  |  |  |  |  |
| 68 | MMP14 | 0.629 | <0.6 |  |  |  |  |  |
| 69 | GALNT5 | 0.629 | <0.6 |  |  |  |  |  |
| 70 | ADAM19 | 0.627 | <0.6 |  |  |  |  |  |
| 71 | SPOCD1 | 0.627 | <0.6 |  |  |  |  |  |
| 72 | ASPN | 0.626 | <0.6 |  |  |  |  |  |
| 73 | CCL26 | 0.625 | <0.6 |  |  |  |  |  |
| 74 | ZEB2 | 0.625 | <0.6 |  |  |  |  |  |
| 75 | **CRISPLD2** | 0.62 | 0.602 |  |  |  |  |  |
| 76 | WIPF1 | 0.62 | <0.6 |  |  |  |  |  |
| 77 | COL6A6 | 0.619 | <0.6 |  |  |  |  |  |
| 78 | GEM | 0.618 | <0.6 |  |  |  |  |  |
| 79 | STARD8 | 0.618 | <0.6 |  |  |  |  |  |
| 80 | DIO2 | 0.617 | <0.6 |  |  |  |  |  |
| 81 | COL6A3 | 0.617 | <0.6 |  |  |  |  |  |
| 82 | C1QTNF6 | 0.615 | <0.6 |  |  |  |  |  |
| 83 | LAMA4 | 0.615 | <0.6 |  |  |  |  |  |
| 84 | CHST11 | 0.611 | <0.6 |  |  |  |  |  |
| 85 | **LOX** | 0.609 | 0.608 |  |  |  |  |  |
| 86 | COL1A2 | 0.609 | <0.6 |  |  |  |  |  |
| 87 | ITGBL1 | 0.608 | <0.6 |  |  |  |  |  |
| 88 | PLAUR | 0.608 | <0.6 |  |  |  |  |  |
| 89 | ASAM | 0.607 | <0.6 |  |  |  |  |  |
| 90 | ALDH1A3 | 0.605 | <0.6 |  |  |  |  |  |
| 91 | PDLIM3 | 0.605 | <0.6 |  |  |  |  |  |
| 92 | COL8A1 | 0.604 | <0.6 |  |  |  |  |  |
| 93 | FLJ35880 | 0.604 | <0.6 |  |  |  |  |  |
| 94 | TNN | 0.604 | <0.6 |  |  |  |  |  |
| 95 | CST7 | 0.603 | <0.6 |  |  |  |  |  |
| 96 | KIAA1949 | 0.603 | <0.6 |  |  |  |  |  |
| 97 | CLEC2B | 0.603 | <0.6 |  |  |  |  |  |
| 98 | CHSY-2 | 0.602 | <0.6 |  |  |  |  |  |
| 99 | TRPV2 | 0.602 | <0.6 |  |  |  |  |  |
| 100 | **SULF1** | 0.602 | 0.603 |  |  |  |  |  |
| 101 | SCG2 | 0.602 | <0.6 |  |  |  |  |  |
| 102 | NNMT | 0.601 | <0.6 |  |  |  |  |  |
| 103 | PCOLCE | 0.601 | <0.6 |  |  |  |  |  |
| 104 | SERPINE1 | 0.601 | <0.6 |  |  |  |  |  |
